# Health dashboard for information management in cervical cancer screening^*^

**DOI:** 10.1590/1518-8345.7084.4446

**Published:** 2025-01-31

**Authors:** Adriana Aparecida Paz, Alloma Christine de Madureira Paula, Ananda Miranda de Lima, Gisele Lopes Castro, Mayara Casagrande Batista da Silva, Lunara Teles Silva

**Affiliations:** 1 Universidade Federal de Ciências da Saúde de Porto Alegre, Porto Alegre, RS, Brazil.; 2 Secretaria Municipal de Saúde de Prudentópolis, Atenção Primária à Saúde, Prudentópolis, PR, Brazil.; 3 Secretaria Municipal de Saúde de Borba, Vigilância em Saúde, Borba, AM, Brazil.; 4 Universidade Federal de Goiás, Goiás, GO, Brazil.

**Keywords:** Uterine Cervical Neoplasms, Papanicolaou Test, Primary Health Care, Information Products and Services, Health Information Management, Nursing

## Abstract

**Objective:**

to create a digital health dashboard for information management in the planning, monitoring, and evaluation of cervical cancer screening.

**Method*:**

the study developed a technological production based on User-Centered Design, using fictitious data from cytopathological exams performed by women between 25 and 64 years old in Primary Health Care. The study complied with the requirements regarding copyright, ethics, and data protection.

**Results:**

the model was created on the Looker Studio^®^ platform. The developed health dashboard is intended for use by nurses, professionals, and health managers. The dashboard’s usability simulations were carried out in fictitious cities. The dashboard optimizes access to information management in near real-time and presents a comprehensive health situation for health planning, monitoring, and evaluation. The model is reusable, which makes it a powerful tool for opportunistic and organized screening activities in the context of Primary Health Care.

**Conclusion*:**

the health dashboard model as an information management tool allows nurses, professionals and health managers to make decisions to improve cervical cancer screening.

## Highlights

**(1)** The health dashboard is intended for use by nurses and health professionals.

**(2)** The information management dashboard can be used for planning, monitoring and evaluation.

**(3)** The dashboard optimizes access to information management in near real time.

**(4)** The dashboard provides a comprehensive view of the status of cytopathological exams performed.

**(5)** The dashboard can be reused with real quarterly reports issued by Brazilian Health Information System for Primary Care (SISAB).

## Introduction

Cervical cancer is a morbidity of great epidemiological and social relevance, given its high incidence and mortality among women. In Brazil, in 2022, an estimated rate of 15.38 cases of this cancer was observed for every 100,000 women aged 25 to 64. However, the incidence varies in the different regions of Brazil, with rates ranging from 8.61 to 26.24 cases per 100,000 women per year^(1-2)^.

Regional disparities are also reflected in the performance of cytopathological examinations. Between 2007 and 2013, approximately 32.3 million women underwent the examination in Brazil. The North Region had a ratio of exams per woman of 1.59, while in the Southeast and Central-West Regions, the proportion was 1.82. This reveals differences in the coverage and frequency of cervical cancer screening in the Unified Health System (SUS – acronym for *Sistema Unificado de Saúde*, in Portuguese), according to the region^(3)^.

Currently, the *Previne Brasil* program, in addition to implementing a new financing modality in the SUS, it also aims to increase accessibility for users and improve the quality of care in Primary Health Care (PHC). One of the program’s goals is the proportion of women between 25 and 64 years old who underwent cytopathological exams in the last three years, in relation to the total number of women in the same age group and territory^(2-5)^. This indicator as goal 4 allows cervical cancer screening through the collection of cytopathological exams in asymptomatic women. Evaluation of the collected material can detect pre-cancerous lesions (abnormal cells) and early stages of cancer (preclinical cancer). This test is widely recognized for its cost-effectiveness and its ability to reduce premature mortality among women of childbearing age^(2,5-6)^.

Cervical cancer screening can be opportunistic or organized. In the opportunistic approach, the woman seeks the test on her own or it is offered by the health professional during another consultation. Organized screening, on the other hand, includes actions coordinated by nurses and PHC professionals, such as monitoring and actively seeking out women^(2,4,7-8)^.

In the complex scenario of epidemiology and the organization of health services, the health indicator referring to the number of women who underwent the cytopathological test reflects the health conditions of a population in a delimited area in a given period. Thus, it provides important information for the planning, management and evaluation of health services, in addition to supporting population survey studies^(6,9-12)^.

The adoption of Information and Communication Technologies (ICTs) and digital transformation in healthcare can facilitate and improve healthcare by introducing new approaches to health-promoting strategies, as well as contributing to the understanding of health patterns in local, municipal, state and national levels^(4,13-16)^. Since 2017, the Digital Health Strategy (DHS) has aimed to improve the quality and increase the accessibility of healthcare services, as well as to improve the flow of information to support clinical and management decisions in healthcare^(17)^.

With the advent of the pandemic scenario and the importance of information management in near real time due to Coronavirus Disease-2019 (COVID-19), the DHS has been consolidating itself through the e-Health platform, integrated with SUS, providing information to benefit users, professionals, managers and healthcare institutions. The initiative aims to foster innovation and drive digital transformation in healthcare processes, while improving governance in the use of information and technological solutions in digital health^(17)^.

DHS seeks to identify and integrate technological resources into health practices to assist management processes, consolidating and disseminating safe and accessible information for health professionals in SUS^(13-17)^. Among the tools, digital health and management panels stand out, which use software or platforms to explore indicators of interest, that is, to monitor data sets, presenting information for health decision-making^(15,18)^. During the COVID-19 pandemic, these digital health panels have proven to be highly effective in the agile and efficient analysis of large volumes of data^(19-20)^.

Real-time information visualization is essential for continuous monitoring of health care^(21-23)^, allowing access to updated data for decision-making in situations that require immediate interventions. This drives digital transformation in health and contributes to improving results in PHC care.

This study is unprecedented in exploring the monitoring of cervical cancer screening through quarterly reports generated by the Brazilian Health Information System for Primary Care (SISAB), displayed on a digital panel in the health unit. No other studies were found in the literature that specifically address the monitoring of cervical cancer screening, although there are studies focused on child and neonatal health^(22-23)^.

Based on SISAB reports for goal 4, six pieces of information are made available, with one called “Present in the Numerator” standing out, which indicates, with the answer “yes”, the women who underwent the cytopathological exam in the last three years. However, the report structure is not user-friendly, as PHC professionals find it difficult to observe the indicator, and using it without proper understanding of the SISAB report compromises the planning, monitoring and evaluation of the cytopathological exams performed.

This study proposes the use of technology in the analysis and monitoring of a *Previne Brasil* health indicator, thus offering a significant contribution to the management and decision-making in PHC. Innovation is characterized by the incorporation of technology into the work process of nurses and health professionals with a view to consolidating and disseminating reliable information and facilitating the planning of more assertive strategies in PHC.

Therefore, the central question of the study is: how can we develop a digital health solution to support the management of information for nurses, health professionals and health managers in the planning, monitoring and evaluation of cytopathological exams for women aged between 25 and 64 years?

Taking into account the epidemiological and social context of cervical cancer, the indicator of the proportion of women who underwent cytopathological examination in PHC in the last three years, as well as the opportunity to apply digital transformation to improve information management in health, the objective of this study was to create a digital health dashboard for information management in the planning, monitoring and evaluation of cervical cancer screening.

## Method

### Study design

This study adopts a methodological approach focused on the technological development of a product in digital health area aimed at nurses, health professionals, and health managers. The focus of the product is to support care and management decisions in the planning, monitoring and evaluation of the indicator of the proportion of cytopathological exams in PHC. The chosen methodology was User-Centered Design (UCD), which prioritizes usability and user experience (UX)^(24)^.

UCD involves an agile development process, aimed at creating intelligent systems that solve problems efficiently, with an emphasis on the requirements from the point of view of the user who will use the technology^(24)^. This process consists of four stages: identification of requirements, creation of solutions, construction of prototypes and evaluation with users^(24-25)^. The last stage is iterative and incremental, evaluating smaller parts as development progresses until reaching the final product. Based on the UX of the authors, professionals and academics in the areas of nursing and pharmacy, and based on their extensive experiences at different levels of health care and in the field of education, this study facilitated the acquisition of skills in using the platform Looker Studio^®(26)^.

### Study setting and period

To develop the digital health dashboard model, a database was structured with information from three fictitious municipalities, chosen according to Brazilian sociodemographic characteristics. These cities were called *Alegria do Norte*, *Gentil Flores* and *Nova Felicidade*, representing different population sizes and similar to Brazilian cities, with up to 10,000, 50,000 and more than 100,000 women, respectively.

To ensure that these municipalities did not exist, a search was carried out on the website of the Brazilian Institute of Geography and Statistics (IBGE, its acronym in Portuguese), which lists all Brazilian cities^(27)^. The planning and execution of the final product took place over approximately four months, from May to August 2022.

### Participants

The study included a total of 160,000 fictitious female participants, all sexually active and between the ages of 25 and 64. The participants were personalized using the Fake Name Generator™, an advanced tool for creating fictitious names and data in 37 languages and for 31 countries, ideal for generating large volumes of fictitious data for developer testing^(28)^.

In the process, first and last names from diverse origins, including North American, British, German, Japanese and Spanish, were selected and organized into five individual spreadsheets generated by the Fake Name Generator™. The compilation of these spreadsheets produced a unique database containing 7,781 first names, 16,860 last names, middle initials from A-Z and a range of birth dates between 07/27/1957 and 07/26/1997, covering ages from 25 to 64.

### Study procedures and variables

The data sources used to compose the digital health dashboard model were based on three fictitious four-monthly reports from SISAB, related to the first four months of 2022, which contain data on women between the ages of 25 and 64, of different population sizes. The structure of the fictitious databases was aligned with the SISAB report format, including six columns: “Name”, Individual Taxpayer Registry (CPF) “CPF”, National SUS Card (CNS) “CNS”, “Date of Birth”, “Last collection” and “Present in the Numerator”. This last column indicates whether the woman underwent the cytopathological test collection in the four-monthly period of the report, with the answer “yes” or “no”. Additionally, two new columns were inserted to record “Scheduling” (yes) and “Deadline” (dd/mm/yyyy), totaling eight dimensions (variables). The Looker Studio^®^ platform (https://lookerstudio.google.com/)was used to create the digital health dashboard model^(26)^. Among the various software options available for creating dashboards, Looker Studio^®^ stands out for being a free tool that transforms report data into interactive and easy-to-share dashboards, adaptable to the specific needs of health services^(22-23,26)^. In addition, the digital solution facilitates cloud storage, allowing real-time access as long as there is an Internet connection.

This platform enabled the association and dissociation of data, as well as the creation of new dimensions and filtering resources for information analysis and management. The interface design was based on Color Theory^(29)^ and included typography, logography, and pie, bar, and line charts for data comparison and the use of filters. For the period analyzed, the digital dashboard model used simulated data from the first four months of 2022, including information on new cytopathological exam collections performed, scheduled, or scheduled, according to the fictitious database.

The calculation of the indicator “proportion of women with cytopathological collection in the PHC”^(11)^ considers the following elements: “Numerator” – that is, the number of women aged 25 to 64 who underwent the cytopathological examination in the PHC in the last three years; “Denominator” – number of women in the same age group registered and linked in the same territory in the period analyzed or potential municipal registration multiplied by the percentage of women between 25 and 64 years old, according to the 2020 population estimates of the IBGE; and the “Multiplication factor” – set at 100.

### Analysis of database simulations in the health dashboard model

The simulations were performed on three fictitious databases, formatted in Google Sheets^®^ to represent different population sizes of women between the ages of 25 and 64. Each spreadsheet included all the dimensions (variables) mentioned above, containing fictitious and random sociodemographic data.

The health dashboard model was subjected to usability tests, which involved the insertion of the different fictitious databases during remote meetings and individual sessions. These tests evaluated several aspects, including ease of use, visual appearance, interactive interface and relevance of the digital health dashboard model. During development, we sought to ensure that the digital dashboard would be replicable and reusable by health professionals, especially nurses, when receiving the quarterly reports from SISAB.

### Ethical aspects

This study did not require analysis by the Research Ethics Committee because it involved the development of a technological product. Measures were adopted to ensure compliance with the General Personal Data Protection Law (LGPD, its acronym in Portuguese) No. 13,709/2018^(30)^ and 13,853/2019^(31)^. To this end, quarterly reports were structured using fictitious sociodemographic data of women between 25 and 64 years old, in fictitious municipalities, within the scope of this technological development study of a product in the area of digital health.

## Results

This study addressed the creation of a digital health dashboard model for nurses, health professionals and health managers in PHC, with the purpose of improving the management of information related to the indicator of the proportion of women who underwent cytopathological examination in the last three years. The model was developed to be used in the health unit and reused with real data from the SISAB quarterly reports. The fictitious data sets that served as data source can be accessed at the electronic address https://bit.ly/BDcpPrevine, allowing its use in simulations.

### Organization of the quarterly report in a database (data source)

The SISAB quarterly report, in spreadsheet format, is a source of detailed information for analyzing the indicator addressed in this study. It includes variables related to time, place and person. The data must be standardized to transform this report into a usable database (data source). During processing, only the columns with the information “Name” (column A), “CPF” (column B), “CNS” (column C), “Date of Birth” (column D), “Last Collection” (column E) and “Present in the Numerator” (column F) should be maintained, as well as two more columns should be added: “Scheduling” (column G) and “Appointment” (column H).

**Figure 1:**
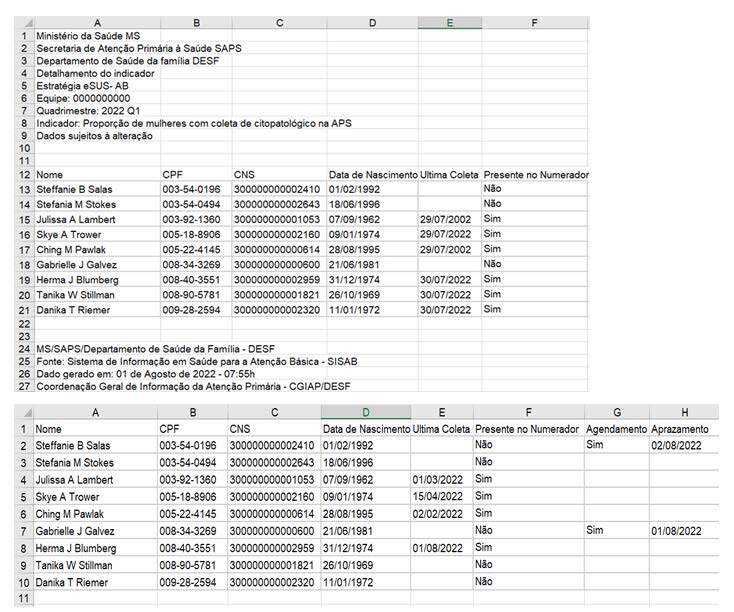
- Treatment of the fictitious quarterly report

The names of the dimensions (variables) of the columns remained unchanged, except for the added columns of “Scheduling” and “Appointment”, used to monitor data related to the performance of cytopathological exams in a timely or organized manner. In this way, the digital dashboard model can consistently recognize the same type of information in any linked database, taking into account differences in upper or lower case letters, as well as the presence or absence of spelling accents. Figure 1 provides an illustration of a fictitious quarterly report similar to those extracted from SISAB.

It is essential that the database be constantly updated by the healthcare professional, especially under the leadership of the nurse, as women undergo the cytopathological exam during the current four-month period. Thus, the healthcare professional in charge must record the date of the “Last Collection” (column E) after the cytopathological exam is performed. In the case of a confirmed appointment (indicated as “yes”) with a scheduled date (dd/mm/yyyy), the “Last Collection” column must be updated after the exam is performed, and the data in the “Schedule” and “Schedule” columns must be removed. This ensures that the results on the health dashboard are always up to date, allowing monitoring of progress towards the goal established by the *Previne Brasil* program.

### Database linking to Looker Studio^®^

**Figure 2:**
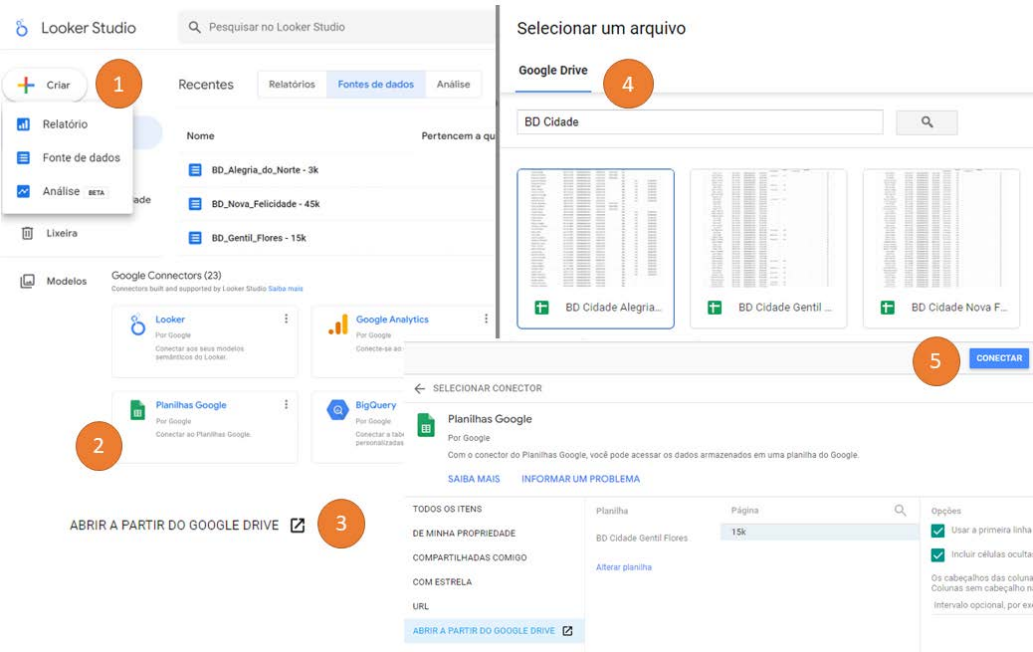
- Linking the database (data source) to Looker Studio^®^

Figure 2 details the database linking process

**Figure 3:**
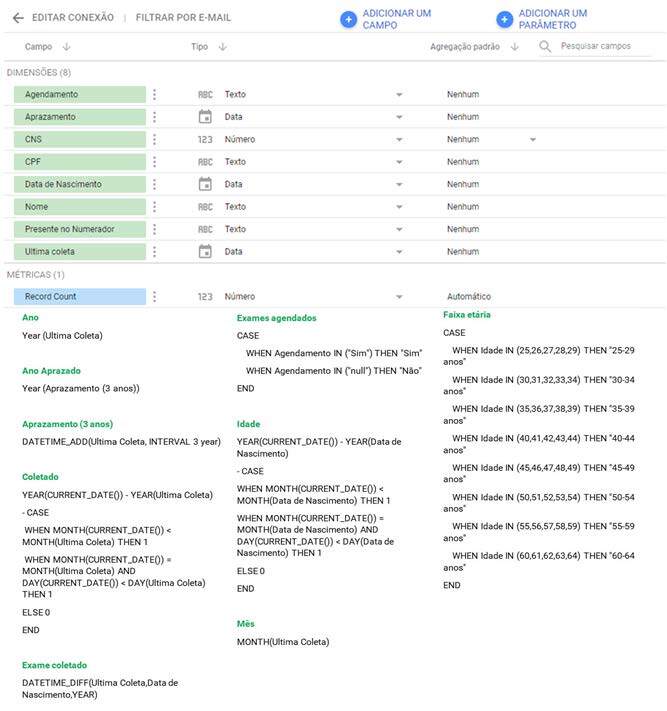
- Dimensions (variables) from the database (data source) to Looker Studio^®^

In step (1) “Data source”, it is possible to establish connections with different types of databases. Then, in step (2), select “Google Sheets” and choose option (3) “Open from Google Drive”. In the subsequent step (4), select the database named “BD_Alegria_do_Norte” and, finally, connect to Looker Studio^®^ in step (5). Figure 3 explores the eight dimensions present in the data source, allowing the creation of new formulas (fx).

The formulas mentioned above provide simple results, associations or dissociations with the aim of consolidating information for the digital health dashboard model. Before using the model, the nine formulas must be incorporated into the data source as new “fields”, resulting in 17 dimensions (variables).

### Creating the health dashboard model

The creation of the interface design for the digital dashboard model was an innovative approach to deal with the complexity of managing information related to the proportion of women who underwent cytopathological examinations. The design incorporated original elements of the complexity and heterogeneity of the indicator’s target audience, highlighting interaction with women as fundamental. Symbols of Feminism (purple), awareness of “Pink October” (pink) and celebration of “International Women’s Day” (lilac) were used, with the application of Color Theories to refine the final interface design.

The digital dashboard model was named Health Information System – Cytopathological (SIS-CP). The results were organized into seven graphs that combine relevant data, allowing a detailed analysis of the health situation related to cytopathological examinations performed in a health unit and/or municipality.

The digital dashboard can be used by nurses, professionals and health managers for information management. It is important to highlight that this panel does not display sensitive data on women, but graphically consolidates data in absolute and relative frequencies and proportions of a fictitious population of the cities simulated in this study.

### Using the health dashboard model

To use the SIS-CP dashboard, you need to connect the database to the model, available at https://bit.ly/SISmeta4Previne. After downloading the model, import it into the healthcare unit’s Looker Studio^®^ to view the data from the fictitious cities mentioned above.

**Figure 4:**
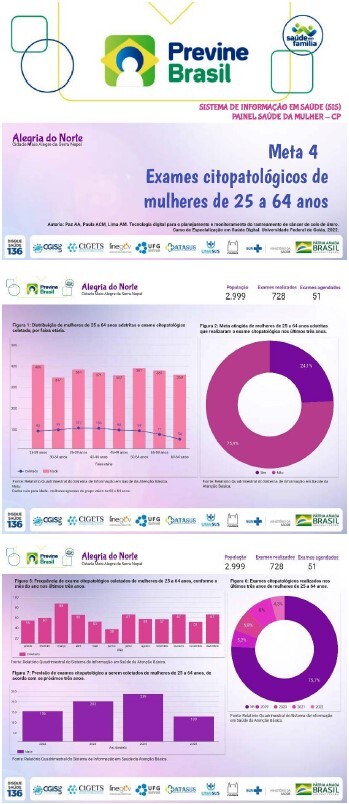
- Digital health dashboard model of the Health Information System – Cytopathology (SIS-CP)

The SIS-CP was composed of four screens. The first screen displays the identification of the healthcare unit and/or municipality, followed by three screens that present the results. The type of graph was selected according to the variable under analysis and its configuration in the data source, considering period, dimension, metric and classification in “Setup”. The dashboard is interactive, allowing information to be explored based on filters applied directly to the graphs. Figure 4 exemplifies the SIS-CP digital dashboard model, using data from the fictitious city of Alegria do Norte.

The name of the fictitious municipality is displayed at the top of the three screens of the SIS-CP dashboard, which can be changed manually. Three graphs are also displayed under the title “Overview” of the Looker Studio^®^ application. These graphs represent: the absolute frequency of women between 25 and 64 years old in the population (“Population”); the absolute frequency of cytopathological tests performed (“Performed exams”); and the absolute frequency of scheduled cytopathological tests (“Scheduled exams”).

On the second screen, the first graph of the SIS-CP dashboard (Figure 4) displays a histogram with a superimposed scatter plot to visualize the distribution of women by age group. The indicator “proportion of women with cytopathological collection in the PHC” is represented in the second graph (Figure 4) by the relative frequency of women who underwent the cytopathological test in the last three years.

On the third screen, the third graph (Figure 4) compares the relative frequency of women by age group with the relative frequency of cytopathological tests collected by age group in the last three years. The fourth graph (Figure 4) shows the relative frequency of cytopathological exams scheduled. The relative frequency of 98.2% in the figure indicates “not recorded (NR)” data in the legend, which occurs due to the absence of the description “yes” in the database for women who underwent cytopathological exam collection and those not scheduled by opportunistic and/or organized screening.

On the last screen, the fifth graph (Figure 4) analyzes the frequency of cytopathological exams collected, distributed by month, seeking to identify the seasonality in the performance of exams in order to plan strategies to increase the collection of cytopathological exams. The sixth graph (Figure 4) shows the relative frequency of cytopathological exams in the last three years, based on the date of the Last Collection recorded. The relative frequency of 75.9% in the graph refers to women without an appointment or schedule for the cytopathological exam. The absence of the date of the actual collection of the exam in the “Last Collection” column of the data source indicates an exam not performed in the observed period (NR). The last graph (Figure 4) projects the number of cytopathological exams to be performed by women every three years, considering the date of the Last Collection.

**Figure 5:**
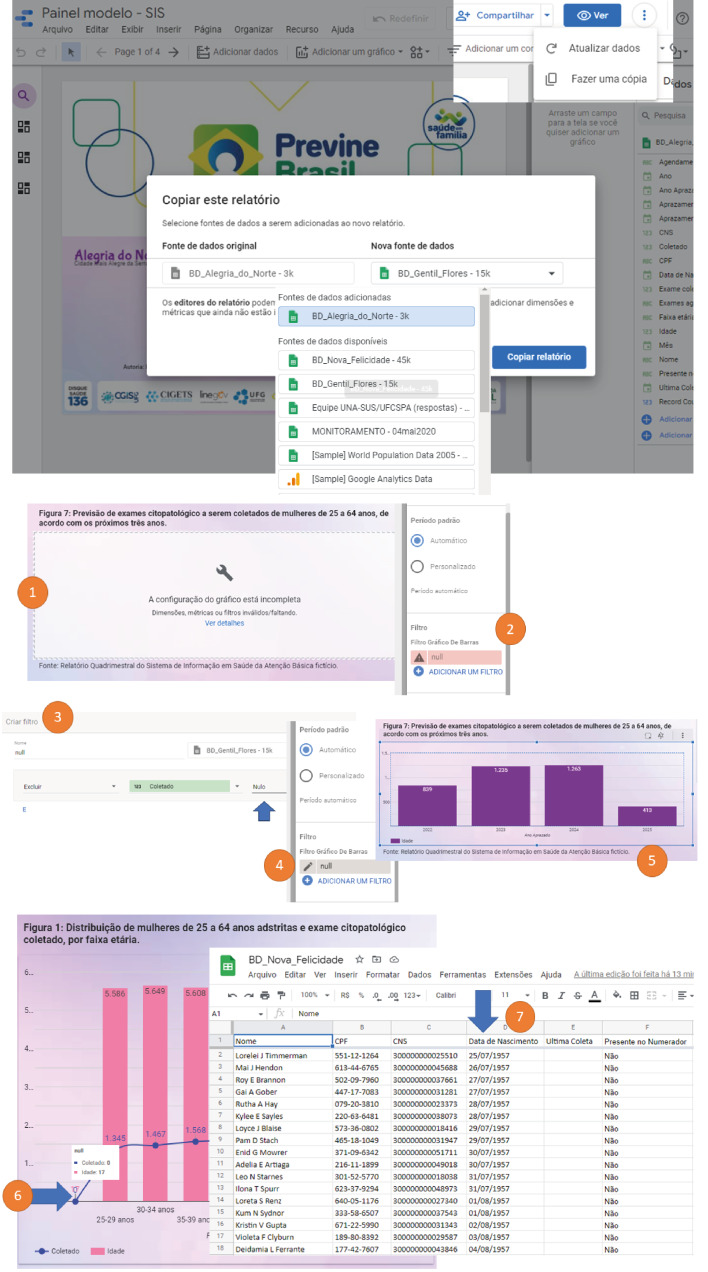
- Database replacement in the digital health dashboard model

To reuse the model, it is essential to replace the database in the “Data Source” of Looker Studio^®^. The SIS-CP digital panel model is available at the electronic address https://bit.ly/BDcpPrevine, allowing simulation with fictitious databases from this study or with real data. If using the database from the SISAB quarterly report, it must be organized to contain the eight basic dimensions mentioned above. Then, it must be created a report for the newly added database. This process must be repeated every four months when the report is updated in SISAB. This step is essential for Looker Studio^®^ to recognize the database as a “Data Source” and integrate the data into the SIS-CP digital panel. Figure 5 details the database replacement process and the minimum adjustments required to (re)use the digital dashboard model. This procedure can be conducted under the leadership of the PHC nurse to ensure the correct functioning of the digital health dashboard while maintaining the protection of sensitive data.

When replacing the “Data Source” in the SIS-CP panel, the platform immediately signals that (1) the chart configuration is incomplete. This occurs due to the (2) filter established for null data in the original database, as exemplified by “BD_Alegria_do_Norte”. Every time a new database is incorporated, such as “BD_Gentil_Flores”, the software identifies this discrepancy between the databases. To correct the expression of the data, it is necessary to: (3) add a new filter in the corresponding field, intended for the new database, maintaining (4) the same name as the filter used previously. After this procedure, (5) the filter will be recognized and the chart planned in the model will be displayed with the correct data. This process ensures that the SIS-CP digital panel works properly with the new “Data Source”, guaranteeing the accuracy and integrity of the information presented. Additionally, in Figure 5, it is worth noting that during the simulation with the “BD_Nova_Felicidade” database, the expression of a small bar before the other age range bars established in the graphs on screens 2 and 3 of the SIS-CP panel was observed (6). These bars emerge due to the continuous updating of data in real time. As evidenced in (7), this occurrence is due to the fact that 17 women no longer fit into the 60 to 64 age range, having turned 65 during the study period. This observation is important when dealing with the evaluation of strategies and the definition of specific priorities for the last age range of women.

The panel model was designed to be reused by health professionals, especially nurses, in their activities in PHC and by municipal management. It provides near-real-time monitoring of the indicator of the proportion of women with cytology collection. It is important to highlight that the use of this SIS-CP digital panel allows the updating of data from cytopathological exams performed as “Last Collection” (dd/mm/yyyy), or the filling in of “Scheduling” (yes) and “Deadline” (dd/mm/yyyy).

## Discussion

The development of the SIS-CP digital dashboard model represents a promising technological innovation for information management, providing a graphical and interactive interface adapted to the current context of PHC units. Previous studies in the area of child and neonatal health^(22-23)^ highlighted functionalities and interface requirements that foster new proposals to improve information management and clinical and administrative decision-making.

Similarly, this study discusses the creation and reuse of the digital dashboard model in the context of PHC, using scenarios of fictitious municipalities for simulations aligned with UX and learning. This model can be applied to real databases from SISAB reports, transforming data into information that is easy to access and interpret.

Data processing and analysis in spreadsheets required robust tools capable of aggregating information in a graphical interface that assists health professionals in decision-making. During the COVID-19 pandemic, this has become even more relevant to provide information to both professionals and the population, in addition to promoting more active participation in health care, using telemonitoring, teleconsultation and telehealth approaches^(16,19-20)^.

In the health area, the implementation of a system that uses a digital health dashboard can be highly beneficial to assist management and care decisions^(15,18,32-33)^. This allows for a detailed analysis of the situation by establishing connections between information, identifying relationships between health problems and their determinants, assessing risks related to diseases and developing future strategies.

This approach is in line with the idea that initiatives to improve cervical cancer screening should be shared^(34)^. Both nurses and other health professionals and managers can use this dashboard for information management; However, the leadership and protagonism of nurses^(35-36)^ stand out in the agility of the team’s response time to define and encourage improvements in the quality of services, aligning with public policies on women’s health and in the management of financial resources in PHC.

In Australia, a study used data from mental health services to make specific adjustments during the COVID-19 pandemic, covering several political-administrative spheres, implementing more effective actions and cooperative agreements for data sharing^(19)^. This example reinforces the importance of digital health dashboards, which offer situational analysis in near real time^(21)^, allowing the proposal of concrete strategies based on epidemiological data.

Successful decision-making is based on the quality of the information accessed by health professionals and managers^(37-38)^. Visual analysis facilitates the analytical reasoning of abstract data through a good graphical interface^(32,39)^. The adoption of digital health dashboards enhances the production of care^(40)^, offering a comprehensive view of patterns, trends, and correlations of results^(38)^ in real time^(8,41)^.

In another study, data extracted from the electronic medical records of hypertensive users in PHC resulted in the construction of a dashboard that demonstrated its usefulness in the workflow of health professionals. By aligning the information with clinical guidelines, this dashboard optimized time and qualified consultations^(39)^. An additional study observed that the dashboard created by Medicare^®^ to promote health equity revealed limited performance in relation to access to health services, allowing for the supervision and prioritization of efforts to improve accessibility^(15)^.

On the other hand, a study that mapped the main data sources for a monitoring and evaluation dashboard of SUS management concluded that there is still a significant challenge due to the fragmentation of information systems in Brazil. However, the researchers relativized this issue, stating that finding instruments and sources of information that are quickly and easily accessible can enable health assessment and monitoring in specific situations^(40)^.

Thus, digital health dashboards demonstrate the ability to analyze and communicate a variety of relevant information^(14,33)^ to nurses and health professionals working in PHC, through the use of SISAB reports. This approach enables dynamic and up-to-date monitoring of the indicator, contributing to information management in PHC.

The digital dashboard model can serve as a basis for guiding the planning, monitoring, and evaluation process of interventions, contributing to improving the accessibility, performance, and quality of health services offered to women. A study evaluating cervical cancer control actions, with data recorded from 2013 to 2020, exemplifies this application^(42)^. Another study on the impact of the COVID-19 pandemic on the performance of cervical cancer screening reinforced the importance of effective actions and strategies to increase adherence and encourage women^(41)^ to take the lead in health care.

This study adopted a social and ethical approach, with the purpose of contributing to the health, education, and society sectors. The digital dashboard model and fictitious databases are available free of charge, allowing simulations with the real four-monthly SISAB reports of any health unit and/or municipality. In this perspective, a survey on the knowledge, attitudes and practices of 170 health professionals belonging to 94 health teams in a municipality in Minas Gerais state emphasized the need for continuing education actions for nurses and doctors in PHC in relation to cervical cancer^(43)^.

Regarding the limitations of using the SIS-CP digital health dashboard, a restriction related to the specific period (four-month period) is observed, requiring that the database be updated and reorganized every four months. Another limitation is the exclusion of data from women under 25 or over 65 years of age, as stipulated by the indicator for cervical cancer screening. In addition, there is a lack of records on the adoption and implementation of new technologies for information management by nurses, health professionals and managers within the SUS.

This study has the potential to instigate future research and the development of mechanisms for planning, monitoring and evaluation in the health area. It also offers opportunities to improve the SIS-CP digital dashboard through UX, aiming to make the information more understandable and accessible to different levels of literacy and health needs. The digital health dashboard model can be applied in simulations during the academic training of nurses and health professionals, expanding the opportunity to improve skills in the use of technologies, such as Looker Studio^®^, to create and improve new digital health dashboards.

## Conclusion

The study highlights the relevance and innovation provided by the creation of a digital health dashboard model (SIS-CP), an advanced digital solution for managing information in PHC. This model stands out for its interactive graphical interface and its ability to provide nurses, professionals and health managers with an effective tool to plan health care for women aged 25 to 64, monitor the performance of cytopathological exams, screen for cervical cancer and assess accessibility and quality of care through the indicator for achieving the target in the *Previne Brasil* program.

The adoption of technology in daily practices allows professionals to make personalized plans that meet the specific needs of the female population in a delimited region. Thus, the tool not only supports decision-making, information management and financing of the SUS, but also contributes to increasing the number of women who undergo cytopathological collection. With this preventive action, it is possible to treat lesions early, increasing survival and reducing premature deaths caused by cervical cancer. Therefore, the SIS-CP digital panel stands out as an important ally in screening for uterine cancer and promoting women’s health.
