# Supplementary material for: Health dashboard for information management in cervical cancer screening
Source: Rev Lat Am Enfermagem. 2025 Jan 31;33:e4446. doi: 10.1590/1518-8345.7084.4446 (PMC11789719; doi:10.1590/1518-8345.7084.4446)
Supplement: Translation

# Translation: Panel de salud para la gestión de la información en el rastreo del cáncer de cuello uterino^*^

**DOI:** 10.1590/1518-8345.7084.4445

**Authors:** Adriana Aparecida Paz, Alloma Christine de Madureira Paula, Ananda Miranda de Lima, Gisele Lopes Castro, Mayara Casagrande Batista da Silva, Lunara Teles Silva

**Affiliations:** 1 Universidade Federal de Ciências da Saúde de Porto Alegre, Porto Alegre, RS, Brasil.; 2 Secretaria Municipal de Saúde de Prudentópolis, Atenção Primária à Saúde, Prudentópolis, PR, Brasil.; 3 Secretaria Municipal de Saúde de Borba, Vigilância em Saúde, Borba, AM, Brasil.; 4 Universidade Federal de Goiás, Goiás, GO, Brasil.

**Keywords:** Neoplasias del Cuello Uterino, Prueba de Papanicolaou, Atención Primaria de Salud, Productos y Servicios de Información, Gestión de la Información en Salud, Enfermería

## Abstract

**Objetivo:**

crear un panel digital de salud para la gestión de la información en la planificación, monitoreo y evaluación del rastreo del cáncer de cuello uterino.

**Método:**

el estudio desarrolló una producción tecnológica fundamentada en el Diseño Centrado en el Usuario, utilizando datos ficticios de exámenes citopatológicos realizados por mujeres entre 25 y 64 años en la Atención Primaria de Salud. El estudio respetó las exigencias en cuanto a derechos de autor, éticos y de protección de datos.

**Resultados:**

la modelación se realizó en la plataforma *Looker Studio* ^®^. El panel de salud desarrollado está destinado al uso de enfermeros, profesionales y gestores de salud. Se realizaron simulaciones de usabilidad del panel entre ciudades ficticias, optimizando el acceso a la gestión de la información en tiempo casi real y presentando una visión integral de la situación de salud para la planificación, monitoreo y evaluación en salud. El modelo es reutilizable, lo que lo convierte en una herramienta potente para actividades de rastreo oportunístico y organizado en el contexto de la Atención Primaria de Salud.

**Conclusión:**

el modelo de panel de salud como herramienta de gestión de la información permite que enfermeros, profesionales y gestores de salud tomen decisiones para mejorar el rastreo del cáncer de cuello uterino.

## Destacados

**(1)** El panel de salud está destinado al uso de enfermeros y profesionales de la salud.

**(2)** El panel de gestión de la información puede ser utilizado en la planificación, monitoreo y evaluación.

**(3)** El panel optimiza el acceso a la gestión de la información en tiempo casi real.

**(4)** El panel propone una visión integral de la situación de los exámenes citopatológicos realizados.

**(5)** El panel es reutilizable con informes cuatrimestrales reales emitidos por el Sistema de Información en Salud para la Atención Básica (SISAB) brasileño.

## Introducción

El cáncer de cuello uterino es una morbilidad de gran relevancia epidemiológica y social, dada su alta incidencia y mortalidad entre las mujeres. En Brasil, en 2022, se estimó una tasa de 15,38 casos de este cáncer por cada 100 mil mujeres de 25 a 64 años. Sin embargo, esta incidencia varía en las diferentes regiones brasileñas, con tasas que oscilan entre 8,61 y 26,24 casos por 100 mil mujeres al año^(1-2)^.

Las disparidades regionales también se reflejan en la realización del examen citopatológico. Entre 2007 y 2013, aproximadamente 32,3 millones de mujeres realizaron el examen en Brasil. La razón de exámenes por mujer fue de 1,59 en la Región Norte, mientras que en las regiones Sudeste y Centro-Oeste, la proporción fue de 1,82, revelando diferencias en la cobertura y frecuencia del rastreo del cáncer de cuello uterino en el Sistema Único de Salud (SUS) brasileño, según la región^(3)^.

Actualmente, el programa *Previne* *Brasil*, además de implementar una nueva modalidad de financiamiento en el SUS, tiene como objetivo ampliar la accesibilidad de las personas usuarias e incrementar la calidad de la atención en la Atención Primaria de Salud (APS). Una de las metas del programa es la proporción de mujeres entre 25 y 64 años que realizaron exámenes citopatológicos en los últimos tres años, en relación con el total de mujeres en la misma franja etaria y territorio^(2-5)^. Este indicador, como meta número 4, permite rastrear el cáncer de cuello uterino mediante la recogida de los examenes citopatológicos en mujeres asintomáticas. La evaluación del material recolectado puede detectar precozmente lesiones precancerosas (células anormales) y estadios iniciales de cáncer (cáncer preclínico). Este examen es ampliamente reconocido por su relación costo-efectividad y por su capacidad de reducir la mortalidad prematura entre mujeres en edad fértil^(2,5-6)^.

El rastreo del cáncer de cuello uterino puede ser oportunista u organizado. En el enfoque oportunista, la mujer busca el examen por su cuenta o este es ofrecido por el profesional de salud durante otra consulta. El rastreo organizado, por su parte, abarca acciones coordinadas por enfermeros y profesionales de la APS, como el monitoreo y la búsqueda activa de las mujeres^(2,4,7-8)^.

En el complejo escenario de la epidemiología y la organización de los servicios de salud, el indicador de salud referente al número de mujeres que realizaron el examen citopatológico refleja las condiciones de salud de una población en un área delimitada en un período determinado. Así, este proporciona información importante para la planificación, gestión y evaluación de los servicios de salud, además de respaldar estudios de encuestas poblacionales^(6,9-12)^.

La adopción de Tecnologías de la Información y Comunicación (TICs) y la transformación digital en la salud pueden facilitar y mejorar la atención a la salud mediante la introducción de nuevos enfoques para estrategias de promoción de la salud, así como contribuir a la comprensión de los patrones de salud a niveles local, municipal, estatal y nacional^(4,13-16)^. Desde 2017, la Estrategia de Salud Digital (ESD) tiene el propósito de mejorar la calidad y ampliar la accesibilidad de los servicios de salud, así como perfeccionar el flujo de información para apoyar las decisiones clínicas y de gestión en salud^(17)^.

Con el advenimiento de la pandemia y la importancia de la gestión de la información en tiempo casi real debido a la enfermedad por Coronavirus-2019 (COVID-19), la ESD se ha consolidado mediante la plataforma e-Salud, integrada al SUS, proporcionando información para beneficiar a usuarios, profesionales, gestores e instituciones de salud. Esta iniciativa tiene como objetivo fomentar la innovación e impulsar la transformación digital en los procesos de salud, al mismo tiempo que mejora la gobernanza en el uso de la información y soluciones tecnológicas en salud digital^(17)^.

La ESD busca identificar e integrar recursos tecnológicos a la práctica de salud para auxiliar en los procesos de gestión, consolidando y difundiendo información segura y accesible para los profesionales de salud en el SUS^(13-17)^. Entre las herramientas, destacan los paneles digitales de salud y de gestión, que utilizan *software* o plataformas para explorar indicadores de interés, es decir, monitorear conjuntos de datos, presentando información para la toma de decisiones en salud^(15,18)^. Durante la pandemia de COVID-19, estos paneles digitales de salud demostraron ser altamente eficaces en el análisis ágil y eficiente de grandes volúmenes de datos^(19-20)^.

La visualización de información en tiempo real es esencial para el monitoreo continuo de la atención a la salud^(21-23)^, permitiendo el acceso a datos actualizados para la toma de decisiones en situaciones que requieren intervenciones inmediatas. Esto impulsa la transformación digital en salud y contribuye a la mejora de los resultados en la atención en la APS.

Este estudio es pionero al explorar el monitoreo del rastreo del cáncer de cuello uterino por intermedio de informes cuatrimestrales generados por el Sistema de Información en Salud para la Atención Básica (SISAB) brasileño, mostrados en un panel digital en la unidad de salud. No se encontraron otros estudios en la literatura que aborden específicamente el monitoreo del rastreo del cáncer de cuello uterino, aunque existen estudios centrados en la salud infantil y neonatal^(22-23)^.

Con base en informes del SISAB de la meta 4, se proporcionan seis informaciones, destacándose una denominada “Presente en el Numerador”, que indica con la respuesta “sí” a las mujeres que realizaron el examen citopatológico en los últimos tres años. Sin embargo, la estructura del informe no es amigable, ya que los profesionales de la APS encuentran dificultades para observar el indicador de manera que el uso sin la debida comprensión del informe SISAB compromete la planificación, monitoreo y evaluación de los exámenes citopatológicos realizados.

Este estudio propone el uso de tecnología en el análisis y seguimiento de un indicador de salud del *Previne* *Brasil*, ofreciendo así una contribución significativa para la gestión y toma de decisiones en la APS. La innovación se caracteriza por la incorporación de la tecnología en el proceso de trabajo del enfermero y de los profesionales de salud con vistas a consolidar y difundir información segura y facilitar la planificación de estrategias más acertadas en la APS.

Así, la pregunta central del estudio es: ¿cómo desarrollar una solución en salud digital para apoyar la gestión de la información de enfermeros, profesionales y gestores de salud en la planificación, monitoreo y evaluación del examen citopatológico de mujeres entre 25 y 64 años?

Teniendo en cuenta el contexto epidemiológico y social del cáncer de cuello uterino, el indicador de la proporción de mujeres que realizaron el examen citopatológico en la APS en los últimos tres años, así como la oportunidad de aplicar la transformación digital para perfeccionar la gestión de la información en salud, el objetivo de este estudio fue crear un panel digital de salud para la gestión de la información en la planificación, monitoreo y evaluación del rastreo del cáncer de cuello uterino.

## Método

### Diseño del estudio

Este estudio adopta un enfoque metodológico centrado en el desarrollo tecnológico de un producto en el área de salud digital, destinado a enfermeros, profesionales y gestores de salud. El enfoque del producto es apoyar decisiones asistenciales y gerenciales en la planificación, monitoreo y evaluación del indicador de proporción de exámenes citopatológicos en la APS. La metodología elegida fue el Diseño Centrado en el Usuario (DCU), que prioriza la usabilidad y la experiencia del usuario (UX)^(24)^.

El DCU implica un proceso ágil de desarrollo, orientado a la creación de sistemas inteligentes que resuelvan problemas de manera eficiente, con énfasis en los requisitos desde el punto de vista del usuario que utilizará la tecnología^(24)^. Este proceso consta de cuatro etapas: identificación de requisitos, creación de soluciones, construcción de prototipos y evaluación con usuarios^(24-25)^. La última etapa es iterativa e incremental, evaluando partes menores a medida que avanza el desarrollo hasta alcanzar el producto final.

Basado en la UX de las autoras, profesionales y académicas del área de enfermería y farmacia, y respaldado por sus amplias experiencias en diversos niveles de atención en salud y en el campo de la enseñanza, este estudio facilitó la adquisición de competencias en el uso de la plataforma *Looker Studio* ^®^^(26)^.

### Local y período del estudio

Para desarrollar el modelo de panel digital de salud, se estructuró una base de datos con información de tres municipios ficticios, elegidos de acuerdo con las características sociodemográficas brasileñas. Estos municipios fueron denominados Alegria do Norte, Gentil Flores y Nova Felicidade, representando diferentes tamaños poblacionales y similares a los municipios brasileños, con hasta 10 mil, 50 mil y más de 100 mil mujeres, respectivamente.

Para asegurar la inexistencia de estos municipios, se realizó una consulta en el sitio *web* del *Instituto Brasileiro de Geografia e Estatística* (IBGE), que lista todas las ciudades brasileñas^(27)^. La planificación y la ejecución del producto final ocurrieron a lo largo de aproximadamente cuatro meses, de mayo a agosto de 2022.

### Participantes

El estudio abarca un total de 160 mil participantes ficticias del sexo femenino, todas con vida sexual activa y edades entre 25 y 64 años. La personalización de las participantes se realizó utilizando *Fake Name Generator*™, una herramienta avanzada para crear nombres y datos ficticios en 37 idiomas y para 31 países, ideal para generar grandes volúmenes de datos ficticios para pruebas de desarrolladores^(28)^.

En el proceso, se seleccionaron nombres y apellidos de diversas orígenes, incluyendo norteamericana, británica, alemana, japonesa y española, organizados en cinco hojas de cálculo individuales generadas por *Fake Name Generator*™. La compilación de estas hojas de cálculo produjo una base de datos única, que contiene 7.781 nombres, 16.860 apellidos, iniciales del segundo nombre de A-Z y un intervalo de fechas de nacimiento entre el 27/07/1957 y el 26/07/1997, abarcando edades de 25 a 64 años.

### Procedimientos y variables del estudio

Las fuentes de datos utilizadas para componer el modelo de panel digital de salud se basaron en tres informes cuatrimestrales ficticios del SISAB, relativos al primer cuatrimestre de 2022, que contienen datos de mujeres entre 25 y 64 años, de diferentes tamaños poblacionales. La estructura de las bases de datos ficticias se alineó con el formato del informe del SISAB, incluyendo seis columnas: “Nombre”, *Cadastro de Pessoa Física* (CPF) “CPF”, *Cartão Nacional do SUS* (CNS) “CNS”, “Fecha de Nacimiento”, “Última recolección” y “Presente en el Numerador”. Esta última columna indica si la mujer realizó la recolección del examen citopatológico en el período cuatrimestral del informe, con respuesta “sí” o “no”. Adicionalmente, se insertaron dos nuevas columnas para registrar “Agendamiento” (sí) y “Programación” (dd/mm/aaaa), totalizando ocho dimensiones (variables).

La plataforma *Looker Studio* ^®^ (https://lookerstudio.google.com/) fue utilizada para la creación del modelo de panel digital de salud^(26)^. Entre las diversas opciones de *software* disponibles para la creación de paneles, *Looker Studio* ^®^ se destaca por ser una herramienta gratuita que transforma datos de informes en paneles interactivos y de fácil compartición, adaptables a las necesidades específicas de los servicios de salud^(22-23,26)^. Además, la solución digital facilita el almacenamiento en la nube, permitiendo el acceso en tiempo real siempre que haya conexión a Internet.

Esta plataforma permitió la asociación y disociación de datos, así como la creación de nuevas dimensiones y recursos de filtrado para el análisis y gestión de la información. El diseño de la interfaz se fundamentó en la Teoría del Color^(29)^ e incluyó elementos de tipografía, logografía y gráficos de pastel, barras y líneas, para la comparación de datos y el uso de filtros. Para el período analizado, el modelo de panel digital utilizó datos simulados del primer cuatrimestre de 2022, incluyendo información sobre nuevas recolecciones de exámenes citopatológicos realizadas, agendadas o programadas, conforme a la base de datos ficticia.

El cálculo del indicador “proporción de mujeres con recolección de citopatológico en la APS”^(11)^ considera los siguientes elementos: “Numerador” – el número de mujeres, entre 25 y 64 años, que realizaron el examen citopatológico en la APS en los últimos tres años; “Denominador” – número de mujeres en la misma franja etaria registradas y vinculadas en el mismo territorio en el período analizado o potencial de registro municipal multiplicado por el porcentaje de mujeres entre 25 y 64 años según estimaciones poblacionales de 2020 del IBGE; y el “Factor de multiplicación” – fijado en 100.

### Análisis de las simulaciones de las bases de datos en el modelo de panel de salud

Las simulaciones se realizaron en tres bases de datos ficticias, formateadas en *Google Sheets* ^®^ para representar diferentes tamaños poblacionales de mujeres entre 25 y 64 años. Cada hoja de cálculo incluía todas las dimensiones (variables) mencionadas anteriormente, conteniendo datos sociodemográficos ficticios y aleatorios.

El modelo de panel de salud fue sometido a pruebas de usabilidad, que involucraron la inserción de las diferentes bases de datos ficticias durante reuniones remotas y sesiones individuales. Estas pruebas evaluaron diversos aspectos, incluyendo la facilidad de uso, la apariencia visual, la interfaz interactiva y la relevancia del modelo de panel digital de salud. Durante el desarrollo, se procuró asegurar que el panel digital fuera replicable y reutilizable por profesionales de salud, especialmente enfermeros, al recibir los informes cuatrimestrales del SISAB.

### Aspectos éticos

Este estudio no requirió la evaluación del Comité de Ética en Investigación por tratarse del desarrollo de un producto tecnológico. Se adoptaron medidas para asegurar la conformidad con la Ley General de Protección de Datos Personales (LGPD) nº 13.709/2018^(30)^ y 13.853/2019^(31)^. Para ello, los informes cuatrimestrales se estructuraron utilizando datos sociodemográficos ficticios de mujeres entre 25 y 64 años en municipios ficticios en el ámbito de este estudio de desarrollo tecnológico de un producto en el área de salud digital.

## Resultados

El presente estudio abordó la creación del modelo de panel digital de salud destinado a enfermeros, profesionales y gestores en la APS, con el propósito de mejorar la gestión de información relacionada con el indicador de la proporción de mujeres que realizaron el examen citopatológico en los últimos tres años. Este modelo fue desarrollado para ser utilizado en la unidad de salud y reutilizado con datos reales de los informes cuatrimestrales del SISAB. Los conjuntos de datos ficticios que sirvieron como fuente de datos pueden ser accedidos en el enlace electrónico https://bit.ly/BDcpPrevine, permitiendo su uso en simulaciones.

### Organización del informe cuatrimestral en una base de datos (fuente de datos)

El informe cuatrimestral del SISAB, en formato de hoja de cálculo, es una fuente de información detallada para el análisis del indicador abordado en este estudio. Este informe abarca variables relacionadas con el tiempo, lugar y persona. Para transformar este informe en una base de datos utilizable (fuente de datos), es necesario estandarizar los datos. Durante este proceso de tratamiento, deben mantenerse solo las columnas con la información “Nombre” (columna A), “CPF” (columna B), “CNS” (columna C), “Fecha de Nacimiento” (columna D), “Última Recolección” (columna E) y “Presente en el Numerador” (columna F). Además, deben añadirse dos columnas más: “Agendamiento” (columna G) y “Programación” (columna H).

Los nombres de las dimensiones (variables) de las columnas permanecieron inalterados, excepto por las columnas añadidas de “Agendamiento” y “Programación”, utilizadas para monitorear datos referentes a la realización de los exámenes citopatológicos de manera oportuna u organizada. De esta manera, el modelo de panel digital puede reconocer de manera consistente el mismo tipo de información en cualquier base de datos vinculada, considerando las diferencias de letras mayúsculas o minúsculas, así como la presencia o ausencia de acentuación ortográfica. La Figura 1 ofrece una ilustración de un informe cuatrimestral ficticio similar a los extraídos del SISAB.

**Figura 1:** - Tratamiento del informe cuatrimestral ficticio

Es fundamental que la base de datos sea actualizada constantemente por el profesional de salud, especialmente bajo la dirección del enfermero, a medida que las mujeres realizan el examen citopatológico durante el cuatrimestre en curso. Así, el profesional de salud responsable debe registrar la fecha de la “Última Recolección” (columna E) tras la realización del examen citopatológico. En caso de agendamiento confirmado (indicado como “sí”) con una fecha de programación (dd/mm/aaaa), la columna “Última Recolección” debe actualizarse después de la realización del examen, y los datos de las columnas “Agendamiento” y “Programación” deben ser eliminados. Esto asegura que los resultados en el panel de salud se mantengan siempre actualizados, permitiendo monitorear el progreso hacia la meta establecida por el programa *PrevineBrasil*.

### Vinculación de la base de datos a *Looker Studio* ^®^

La Figura 2 detalla el proceso de vinculación de la base de datos.

**Figura 2:** - Vinculación de la base de datos (fuente de datos) a *Looker Studio* ^®^

En la etapa (1) “Fuente de datos”, es posible establecer conexiones con diversos tipos de bases de datos. A continuación, en la etapa (2), se selecciona “Hojas de cálculo de *Google*” y se elige la opción (3) “Abrir desde *Google Drive*”. En la etapa siguiente (4), se selecciona la base de datos denominada “BD_Alegria_do_Norte” y, finalmente, se realiza la conexión con *Looker Studio* ^®^ en la etapa (5). La Figura 3 explora las ocho dimensiones presentes en la fuente de datos, permitiendo la elaboración de nuevas fórmulas (fx).

**Figura 3:** - Dimensiones (variables) de la base de datos (fuente de datos) en *Looker Studio* ^®^

Las fórmulas mencionadas anteriormente ofrecen resultados simples, asociaciones o disociaciones con el objetivo de consolidar información para el modelo de panel digital de salud. Antes de la utilización del modelo, estas nueve fórmulas deben ser incorporadas a la fuente de datos como nuevos “campos”, resultando en 17 dimensiones (variables).

### Creación del modelo de panel de salud

La creación del diseño de interfaz del modelo de panel digital fue un enfoque innovador para abordar la complejidad de la gestión de la información relacionada con la proporción de mujeres que realizaron el examen citopatológico. El diseño incorporó elementos originales de la complejidad y heterogeneidad del público objetivo del indicador, destacando la interacción con las mujeres como fundamental. Se utilizaron símbolos del Feminismo (morado), la concienciación del “Octubre Rosa” (rosa) y la celebración del “Día Internacional de la Mujer” (lila), aplicando la Teoría del Color para refinar el diseño de la interfaz final.

El modelo de panel digital recibió el nombre de Sistema de Información en Salud – Citopatológico (SIS-CP). Los resultados se organizaron en siete gráficos que combinan datos relevantes, permitiendo un análisis detallado de la situación de salud relacionada con los exámenes citopatológicos realizados en una unidad de salud y/o municipio.

El panel digital puede ser utilizado por enfermeros, profesionales y gestores de salud para la gestión de la información. Es importante destacar que dicho panel no muestra datos sensibles de las mujeres, sino que consolida gráficamente datos en frecuencias absolutas, relativas y proporciones de una población ficticia de las ciudades simuladas en este estudio.

### Uso del modelo de panel de salud

Para utilizar el panel SIS-CP, es necesario conectar la base de datos al modelo, disponible en el siguiente enlace: https://bit.ly/SISmeta4Previne. Después de descargado, el modelo debe ser importado en *Looker Studio* ^®^ de la unidad de salud para visualizar los datos de las ciudades ficticias mencionadas anteriormente.

El SIS-CP está compuesto por cuatro pantallas. La primera pantalla muestra la identificación de la unidad de salud y/o municipio, seguida por tres pantallas que presentan los resultados. El tipo de gráfico se seleccionó de acuerdo con la variable en análisis y su configuración en la fuente de datos, considerando período, dimensión, métrica y clasificación en el *Setup*. El panel es interactivo, permitiendo la exploración de la información en función de los filtros aplicados directamente en los gráficos. La Figura 4 ejemplifica el modelo de panel digital SIS-CP, utilizando datos de la ciudad ficticia de Alegria do Norte.

**Figura 4:** - Modelo de panel digital de salud del Sistema de Información en Salud – Citopatológico (SIS-CP)

En la parte superior de las tres pantallas del panel SIS-CP, se muestra el nombre del municipio ficticio, que puede ser modificado manualmente. También se presentan tres gráficos bajo el título “Visión General” de la aplicación *Looker Studio* ^®^. Estos gráficos representan: la frecuencia absoluta de mujeres entre 25 y 64 años en la población (“Población”); la frecuencia absoluta de exámenes citopatológicos realizados (“Exámenes realizados”); y la frecuencia absoluta de exámenes citopatológicos programados (“Exámenes programados”).

En la segunda pantalla, el primer gráfico del panel SIS-CP (Figura 4) muestra un histograma con una dispersión sobrepuesta para visualizar la distribución de mujeres por grupo de edad. El indicador “proporción de mujeres con recolección de citopatológico en la APS” está representado en el segundo gráfico (Figura 4) por la frecuencia relativa de las mujeres que realizaron el examen citopatológico en los últimos tres años.

En la tercera pantalla, el tercer gráfico (Figura 4) compara la frecuencia relativa de las mujeres por grupo de edad con la frecuencia relativa de exámenes citopatológicos recolectados por grupo de edad en los últimos tres años. El cuarto gráfico (Figura 4) presenta la frecuencia relativa de programaciones de exámenes citopatológicos. La frecuencia relativa del 98,2% en la figura indica un dato “no registrado (NR)” en la leyenda, lo cual ocurre debido a la ausencia de la descripción “sí” en la base de datos para las mujeres que realizaron la recolección del examen citopatológico y aquellas no programadas por el rastreo oportunista y/o organizado.

En la última pantalla, el quinto gráfico (Figura 4) analiza la frecuencia de exámenes citopatológicos recolectados, distribuidos por mes, buscando identificar la estacionalidad en la realización de los exámenes para planear estrategias de incremento en la recolección de exámenes citopatológicos. El sexto gráfico (Figura 4) muestra la frecuencia relativa de la realización del examen citopatológico en los últimos tres años, basada en la fecha de la Última Recolección registrada. La frecuencia relativa del 75,9% en el gráfico se refiere a las mujeres sin programación y sin programación para el examen citopatológico. La ausencia de la fecha de recolección efectiva del examen en la columna “Última Recolección” de la fuente de datos indica un examen no realizado en el período observado (NR). El último gráfico (Figura 4) proyecta la cantidad de exámenes citopatológicos a ser realizados por las mujeres cada tres años, considerando la fecha de la Última Recolección.

Para reutilizar el modelo, es esencial sustituir la base de datos en la “Fuente de datos” de *Looker Studio* ^®^. El modelo de panel digital SIS-CP está disponible en el enlace: https://bit.ly/BDcpPrevine, permitiendo la simulación con bases de datos ficticias de este estudio o con datos reales. En caso de utilizar la base de datos proveniente del informe cuatrimestral del SISAB, ella debe ser organizada de manera que contenga las ocho dimensiones básicas mencionadas anteriormente. Luego, debe crear un informe para la base de datos recién añadida. Este proceso debe ser repetido cada cuatrimestre cuando el informe se actualice en el SISAB. Esta etapa es fundamental para que *Looker Studio* ^®^ reconozca la base de datos como una “Fuente de datos” e integre los datos al panel digital SIS-CP.

La Figura 5 detalla el proceso de sustitución de bases de datos y los ajustes mínimos necesarios para (re)utilizar el modelo de panel digital. Este procedimiento puede ser llevado a cabo bajo la dirección del enfermero en la APS para asegurar el correcto funcionamiento del panel digital de salud, manteniendo la protección de datos sensibles.

**Figura 5:** - Sustitución de base de datos en el modelo de panel digital de salud

Al sustituir la “Fuente de datos” en el panel SIS-CP, la plataforma inmediatamente señala que (1) la configuración del gráfico está incompleta. Esto ocurre debido al (2) filtro establecido para datos nulos (*null*) en la base de datos original, como se ejemplifica con el “BD_Alegria_do_Norte”. Cada vez que se incorpora una nueva base de datos, como por ejemplo el “BD_Gentil_Flores”, el *software* identifica esta discrepancia entre las bases. Para corregir la expresión de los datos, es necesario: (3) añadir un nuevo filtro en el campo correspondiente, destinado a la nueva base de datos, manteniendo (4) el mismo nombre del filtro utilizado anteriormente. Después de este procedimiento, (5) el filtro será reconocido y el gráfico planeado en el modelo se mostrará con los datos correctos. Este proceso asegura que el panel digital SIS-CP funcione adecuadamente con la nueva “Fuente de datos”, garantizando la precisión y la integridad de la información presentada.

Adicionalmente, en la Figura 5, es pertinente resaltar que durante la simulación con la base de datos “BD_Nova_Felicidade”, se observó la (6) expresión de una pequeña barra anterior a las demás barras de grupos etarios establecidos en los gráficos de las pantallas 2 y 3 del panel SIS-CP. Estas barras emergen debido a la actualización continua de los datos en tiempo real. Como se evidenció (7), tal ocurrencia se debe al hecho de que 17 mujeres ya no se encuentran en el grupo etario de 60 a 64 años, habiendo cumplido 65 años durante el período del estudio. Esta observación cobra importancia al tratar la evaluación de estrategias y la definición de prioridades específicas para el último grupo etario de mujeres.

El modelo de panel fue ideado para ser reutilizado por profesionales de salud, sobre todo enfermeros en sus actividades en la APS, así como por la gestión municipal, proporcionando un seguimiento en tiempo casi real del indicador de proporción de mujeres con recolección de citopatológico. Es importante resaltar que el uso de este panel digital SIS-CP permite la actualización de datos de exámenes citopatológicos realizados como “Última Recolección” (dd/mm/aaaa), o el llenado de “Agendamiento” (sí) y “Programación” (dd/mm/aaaa).

## Discusión

El desarrollo del modelo de panel digital SIS-CP representa una innovación tecnológica prometedora para la gestión de la información, proporcionando una interfaz gráfica e interactiva adaptada al contexto actual de las unidades de la APS. Estudios anteriores en el área de salud infantil y neonatal^(22-23)^ destacaron funcionalidades y requisitos de interfaz que fomentan nuevas propuestas para mejorar la gestión de información y la toma de decisiones clínicas y administrativas.

De manera similar, este estudio discute la creación y reutilización del modelo de panel digital en el contexto de la APS, utilizando escenarios de municipios ficticios para simulaciones alineadas con la UX y el aprendizaje. Este modelo puede aplicarse a bases de datos reales provenientes de los informes del SISAB, transformando datos en información de fácil acceso e interpretación.

El procesamiento y análisis de datos en las hojas de cálculo requirieron herramientas robustas capaces de agregar información en una interfaz gráfica que ayude a los profesionales de salud en la toma de decisiones. Durante la pandemia de COVID-19, esto se volvió aún más relevante para poner a disposición información tanto para los profesionales como para la población, además de promover una participación más activa en los cuidados de salud, utilizando enfoques de telemonitoreo, teleconsultas y telesalud^(16,19-20)^.

En el área de la salud, la implementación de un sistema que emplea un panel digital de salud puede ser altamente beneficiosa para asistir en las decisiones gerenciales y asistenciales^(15,18,32-33)^. Esto permite un análisis detallado de la situación al establecer conexiones entre información, identificar relaciones entre problemas de salud y sus determinantes, evaluar riesgos relacionados con enfermedades y elaborar estrategias futuras.

Este enfoque se alinea con la idea de que las iniciativas para mejorar el rastreo del cáncer de cuello uterino deben ser compartidas^(34)^. Tanto enfermeros como otros profesionales y gestores de salud pueden utilizar este panel para la gestión de la información; sin embargo, se destacan el liderazgo y el protagonismo del enfermero^(35-36)^ en la agilidad del tiempo de respuesta del equipo para definir e incentivar mejoras en la calidad de los servicios, alineándose con las políticas públicas de salud de la mujer y en la gestión de recursos financieros en la APS.

En Australia, un estudio utilizó datos de los servicios de salud mental para realizar ajustes puntuales durante la pandemia de COVID-19, abarcando varias esferas político-administrativas, implementando acciones más eficaces y acuerdos cooperativos para compartir datos^(19)^. Este ejemplo refuerza la importancia de los paneles digitales de salud, que ofrecen análisis situacional en tiempo casi real^(21)^, permitiendo la proposición de estrategias concretas basadas en datos epidemiológicos.

El éxito en la toma de decisiones se basa en la calidad de la información accedida por los profesionales y gestores de salud^(37-38)^. El análisis visual facilita el razonamiento analítico de datos abstractos mediante una buena interfaz gráfica^(32,39)^. La adopción de paneles digitales de salud valora la producción de cuidados^(40)^, ofreciendo una visión integral de patrones, tendencias y correlaciones de resultados^(38)^ en tiempo real^(8,41)^.

En otro estudio, los datos extraídos de los registros electrónicos de usuarios hipertensos en la APS resultaron en la construcción de un panel que demostró su utilidad en el flujo de trabajo de los profesionales de salud. Al alinear la información con las directrices clínicas, este panel optimizó el tiempo y mejoró la calidad de las consultas^(39)^. Un estudio adicional observó que el panel creado por *Medicare* ^®^ para promover equidad en salud reveló un desempeño limitado en relación con el acceso a los servicios de salud, permitiendo la supervisión y la priorización de esfuerzos para mejorar la accesibilidad^(15)^.

Por otro lado, un estudio que mapeó las principales fuentes de datos para un panel de monitoreo y evaluación de la gestión del SUS concluyó que todavía existe un desafío significativo debido a la fragmentación de los sistemas de información en Brasil. Sin embargo, los investigadores relativizaron esta cuestión, afirmando que encontrar instrumentos y fuentes de información de acceso rápido y fácil puede viabilizar la evaluación y monitoreo de salud en situaciones específicas^(40)^.

Así, los paneles digitales de salud demuestran la capacidad de analizar y comunicar una variedad de información relevante^(14,33)^ para enfermeros y profesionales de salud que actúan en la APS, mediante el aprovechamiento de los informes del SISAB. Este enfoque permite un monitoreo dinámico y actualizado del indicador, contribuyendo a la gestión de la información en la APS.

El modelo de panel digital puede servir como base para orientar el proceso de planificación, monitoreo y evaluación de intervenciones, contribuyendo a la mejora de la accesibilidad, el desempeño y la calidad de los servicios de salud ofrecidos a las mujeres. Un estudio que evaluó acciones de control del cáncer de cuello uterino con datos registrados de 2013 a 2020 ejemplifica esta aplicación^(42)^. Otro estudio sobre el impacto de la pandemia de COVID-19 en la realización del examen preventivo del cáncer de cuello uterino reforzó la importancia de acciones y estrategias eficaces para aumentar la adhesión e incentivar a las mujeres^(41)^ a asumir el protagonismo en el cuidado de su salud.

Este estudio adoptó un enfoque social y ético, con el propósito de contribuir a los sectores de la salud, la educación y la sociedad. El modelo de panel digital y las bases de datos ficticias están disponibles de forma gratuita, permitiendo simulaciones con los informes cuatrimestrales reales del SISAB de cualquier unidad de salud y/o municipio. En esta perspectiva, una investigación sobre conocimientos, actitudes y prácticas de 170 profesionales de salud pertenecientes a 94 equipos de salud de un municipio de Minas Gerais enfatizó la necesidad de acciones de educación continua para enfermeros y médicos en la APS en relación con el cáncer de cuello uterino^(43)^.

En cuanto a las limitaciones del uso del panel digital de salud SIS-CP, se observa una restricción relacionada con el período específico (cuatrimestre), lo que requiere que la base de datos sea actualizada y reorganizada cada cuatro meses. Otra limitación es la exclusión de datos de mujeres menores de 25 años o mayores de 65 años, según lo estipulado por el indicador para el rastreo del cáncer de cuello uterino. Además, hay una escasez de registros sobre la adopción e implementación de nuevas tecnologías para la gestión de la información por parte de enfermeros, profesionales de salud y gestores en el ámbito del SUS.

Este estudio tiene la capacidad de impulsar futuras investigaciones y el desarrollo de mecanismos destinados a la planificación, monitoreo y evaluación en el área de la salud. También ofrece oportunidades para mejorar el panel digital SIS-CP mediante la UX, con el fin de hacer la información más comprensible y accesible para diferentes niveles de literacidad y necesidades de salud. El modelo de panel digital de salud puede aplicarse en simulaciones durante la formación académica de enfermeros y profesionales del área de la salud, ampliando la oportunidad de mejorar las habilidades en el uso de tecnologías, como *Looker Studio* ^®^, para crear y perfeccionar nuevos paneles digitales de salud.

## Conclusión

El estudio destaca la relevancia y la innovación proporcionadas por la creación de un modelo de panel digital de salud SIS-CP, una solución digital avanzada para la gestión de información en la APS. Este modelo se distingue por su interfaz gráfica interactiva y por la capacidad de ofrecer a enfermeros, profesionales y gestores de salud una herramienta eficaz para planificar la atención de salud de las mujeres entre 25 y 64 años, monitorear la realización del examen citopatológico, rastrear el cáncer de cuello uterino y evaluar la accesibilidad y la calidad asistencial por medio del indicador para alcanzar la meta en el programa *Previne* *Brasil*.

La adopción de tecnología en las prácticas diarias permite a los profesionales un planeamiento personalizado que atiende las necesidades específicas de la población femenina de una región delimitada. Así, esta herramienta no solo apoya la toma de decisiones, la gestión de la información y la financiación del SUS, sino que también contribuye a aumentar el número de mujeres que realizan la recolección del citopatológico. Con esta acción preventiva, es posible tratar lesiones de manera precoz, aumentando la supervivencia y reduciendo las muertes prematuras causadas por el cáncer de cuello uterino. Por lo tanto, el panel digital SIS-CP se destaca como un importante aliado en el rastreo del cáncer de útero y en la promoción de la salud de las mujeres.

# Translation: Painel de saúde para gestão da informação no rastreamento do câncer de colo de útero^*^

**DOI:** 10.1590/1518-8345.7084.4447

**Authors:** Adriana Aparecida Paz, Alloma Christine de Madureira Paula, Ananda Miranda de Lima, Gisele Lopes Castro, Mayara Casagrande Batista da Silva, Lunara Teles Silva

**Affiliations:** 1 Universidade Federal de Ciências da Saúde de Porto Alegre, Porto Alegre, RS, Brasil.; 2 Secretaria Municipal de Saúde de Prudentópolis, Atenção Primária à Saúde, Prudentópolis, PR, Brasil.; 3 Secretaria Municipal de Saúde de Borba, Vigilância em Saúde, Borba, AM, Brasil.; 4 Universidade Federal de Goiás, Goiás, GO, Brasil.

**Keywords:** Neoplasias do Colo do Útero, Teste de Papanicolaou, Atenção Primária à Saúde, Produtos e Serviços de Informação, Gestão da Informação em Saúde, Enfermagem

## Abstract

**Objetivo:**

criar um painel digital de saúde para a gestão da informação no planejamento, monitoramento e avaliação do rastreamento do câncer de colo de útero.

**Método:**

o estudo desenvolveu uma produção tecnológica fundamentada no *Design* Centrado no Usuário, utilizando dados fictícios de exames citopatológicos realizados por mulheres entre 25 e 64 anos na Atenção Primária à Saúde. O estudo respeitou as exigências quanto a direitos autorais, éticos e proteção de dados.

**Resultados:**

a modelagem foi feita na plataforma *Looker Studio* ^®^. O painel de saúde desenvolvido destina-se ao uso de enfermeiros, profissionais e gestores de saúde. Realizaram-se simulações de usabilidade do painel entre cidades fictícias, otimizando o acesso à gestão da informação em tempo quase real e apresentando uma visão abrangente da situação de saúde para o planejamento, monitoramento e avaliação em saúde. O modelo é reutilizável, o que o torna uma ferramenta potente para atividades de rastreamento oportunístico e organizado no contexto da Atenção Primária à Saúde.

**Conclusão:**

o modelo de painel de saúde como ferramenta de gestão da informação permite que enfermeiros, profissionais e gestores de saúde tomem decisões para aprimorar o rastreamento do câncer de colo de útero.

## Destaques

**(1)** O painel de saúde destina-se ao uso de enfermeiros e profissionais de saúde.

**(2)** O painel de gestão da informação pode ser utilizado no planejamento, monitoramento e avaliação.

**(3)** O painel otimiza o acesso à gestão da informação em tempo quase real.

**(4)** O painel propõe visão abrangente da situação dos exames citopatológicos realizados.

**(5)** O painel é reutilizável com relatórios quadrimestrais reais emitidos pelo Sistema de Informação em Saúde para a Atenção Básica (SISAB).

## Introdução

O câncer de colo do útero é uma morbidade de grande relevância epidemiológica e social, dada sua alta incidência e mortalidade entre as mulheres. No Brasil, em 2022, estimou-se uma taxa de 15,38 casos desse câncer para cada 100 mil mulheres de 25 a 64 anos. Contudo, a incidência varia nas diferentes regiões brasileiras, com taxas que oscilam de 8,61 a 26,24 casos por 100 mil mulheres ao ano^(1-2)^.

As disparidades regionais também se refletem na realização do exame citopatológico. Entre 2007 e 2013, aproximadamente 32,3 milhões de mulheres realizaram o exame no Brasil. Observou-se a razão de exames por mulher de 1,59 na Região Norte, ao passo que nas regiões Sudeste e Centro-Oeste, a proporção foi de 1,82, revelando diferenças na cobertura e frequência de rastreamento do câncer de colo de útero no Sistema Único de Saúde (SUS), de acordo com a região^(3)^.

Atualmente, o programa Previne Brasil, além de implementar uma nova modalidade de financiamento no SUS, objetiva ampliar a acessibilidade das pessoas usuárias e incrementar a qualidade da assistência na Atenção Primária à Saúde (APS). Uma das metas do programa é a proporção de mulheres entre 25 a 64 anos que realizaram exames citopatológicos nos últimos três anos, em relação ao total de mulheres na mesma faixa etária e território^(2-5)^. Esse indicador como meta número 4 permite o rastreamento do câncer de colo do útero por meio da coleta de exame citopatológico em mulheres assintomáticas. A avaliação do material coletado pode detectar precocemente lesões pré-cancerígenas (células anormais) e estágios iniciais de câncer (câncer pré-clínico). Esse exame é amplamente reconhecido por sua relação custo-efetividade e por sua capacidade de reduzir a mortalidade prematura entre mulheres em idade fértil^(2,5-6)^.

O rastreamento do câncer de colo de útero pode ser oportunístico ou organizado. Na abordagem oportunística, a mulher busca o exame por conta própria ou ele é oferecido pelo profissional de saúde durante outra consulta. Já o rastreamento organizado engloba ações coordenadas por enfermeiros e profissionais da APS, como o monitoramento e a busca ativa das mulheres^(2,4,7-8)^.

No complexo cenário da epidemiologia e da organização dos serviços de saúde, o indicador de saúde referente ao número de mulheres que realizaram o exame citopatológico reflete as condições de saúde de uma população em área delimitada em um período determinado. Assim, ele fornece informações importantes para o planejamento, gerenciamento e avaliação dos serviços de saúde, além de sustentar estudos de inquéritos populacionais^(6,9-12)^.

A adoção de Tecnologias da Informação e Comunicação (TICs) e a transformação digital na saúde podem facilitar e aprimorar a atenção à saúde por meio da introdução de novas abordagens para estratégias promotoras de saúde, assim como podem contribuir para a compreensão dos padrões de saúde em níveis local, municipal, estadual e nacional^(4,13-16)^. Desde 2017, a Estratégia de Saúde Digital (ESD) possui o propósito de aprimorar a qualidade e ampliar a acessibilidade dos serviços de saúde, bem como aperfeiçoar o fluxo de informações para apoio às decisões clínicas e de gestão na saúde^(17)^.

Com o advento da pandemia e a importância da gestão da informação em tempo quase real pelo *Coronavirus Disease-2019* (COVID-19), a ESD vem se consolidando através da plataforma e-Saúde, integrada ao SUS, disponibilizando informações para beneficiar usuários, profissionais, gestores e instituições de saúde. A iniciativa visa fomentar a inovação e impulsionar a transformação digital nos processos de saúde, ao mesmo tempo em que aprimora a governança no uso de informações e soluções tecnológicas em saúde digital^(17)^.

A ESD busca identificar e integrar recursos tecnológicos à prática de saúde para auxiliar nos processos de gestão, consolidando e disseminando informações seguras e acessíveis para profissionais de saúde no SUS^(13-17)^. Entre as ferramentas, destacam-se os painéis digitais de saúde e de gestão, que utilizam *softwares* ou plataformas para explorar indicadores de interesse, ou seja, monitorar conjuntos de dados, apresentando informações para tomada de decisão em saúde^(15,18)^. Durante a pandemia de COVID-19, esses painéis digitais de saúde provaram ser altamente eficazes na análise ágil e eficiente de grandes volumes de dados^(19-20)^.

A visualização de informações em tempo real é essencial para o monitoramento contínuo da atenção à saúde^(21-23)^, permitindo o acesso a dados atualizados para a tomada de decisões em situações que requerem intervenções imediatas. Isso impulsiona a transformação digital na saúde e contribui para a melhoria dos resultados na assistência na APS.

Este estudo é inédito ao explorar o monitoramento do rastreamento do câncer de colo do útero por meio de relatórios quadrimestrais gerados pelo Sistema de Informação em Saúde para a Atenção Básica (SISAB), exibidos em um painel digital na unidade de saúde. Não foram encontrados outros estudos na literatura que abordem especificamente o monitoramento do rastreamento do câncer de colo de útero, embora existam estudos focados em saúde infantil e neonatal^(22-23)^.

Com base em relatórios do SISAB da meta número 4, disponibilizam-se seis informações, destacando-se uma nominada de “Presente no Numerador”, que indica, com a resposta “sim”, as mulheres que realizaram o exame citopatológico nos últimos três anos. No entanto, a estrutura do relatório não é amigável, pois os profissionais da APS encontram dificuldades para observar o indicador, de forma que o uso sem a devida compreensão do relatório SISAB compromete o planejamento, monitoramento e avaliação dos exames citopatológicos realizados.

Este estudo propõe o uso de tecnologia na análise e acompanhamento de um indicador de saúde do Previne Brasil, oferecendo, dessa forma, uma contribuição significativa para a gestão e a tomada de decisões na APS. A inovação caracteriza-se pela incorporação da tecnologia no processo de trabalho do enfermeiro e dos profissionais de saúde com vistas a consolidar e disseminar informações seguras e facilitar o planejamento de estratégias mais assertivas na APS.

Assim, a pergunta central do estudo é: como desenvolver uma solução em saúde digital para apoiar a gestão da informação de enfermeiros, profissionais e gestores de saúde no planejamento, monitoramento e avaliação do exame citopatológico de mulheres com idade entre 25 e 64 anos?

Levando-se em consideração o contexto epidemiológico e social do câncer de colo de útero, o indicador da proporção de mulheres que realizaram o exame citopatológico na APS nos últimos três anos, bem como a oportunidade de aplicar a transformação digital para aperfeiçoar a gestão da informação na saúde, o objetivo deste estudo foi criar um painel digital de saúde para a gestão da informação no planejamento, monitoramento e avaliação do rastreamento do câncer de colo de útero.

## Método

### Delineamento do estudo

Este estudo adota uma abordagem metodológica centrada no desenvolvimento tecnológico de um produto na área da saúde digital destinado a enfermeiros, profissionais e gestores de saúde. O foco do produto é apoiar decisões assistenciais e gerenciais no planejamento, monitoramento e avaliação do indicador de proporção de exames citopatológicos na APS. A metodologia escolhida foi o *Design* Centrado no Usuário (DCU), que prioriza a usabilidade e a experiência do usuário (UX)^(24)^.

O DCU envolve um processo ágil de desenvolvimento, direcionado à criação de sistemas inteligentes que resolvam problemas de forma eficiente, com ênfase nos requisitos do ponto de vista do usuário que utilizará a tecnologia^(24)^. Esse processo é composto por quatro etapas: identificação de requisitos, criação de soluções, construção de protótipos e avaliação com usuários^(24-25)^. A última etapa é iterativa e incremental, avaliando partes menores conforme o desenvolvimento avança, até alcançar o produto final.

Com base na UX das autoras, profissionais e acadêmicas da área de enfermagem e farmácia, e embasadas em suas amplas vivências em diversos níveis de atenção à saúde e no campo do ensino, este estudo facilitou a aquisição de competências no uso da plataforma *Looker Studio* ^®(26)^.

### Local e período do estudo

Para desenvolver o modelo de painel digital de saúde, foi estruturado um banco de dados com informações de três municípios fictícios, escolhidos de acordo com as características sociodemográficas brasileiras. Esses municípios foram denominados Alegria do Norte, Gentil Flores e Nova Felicidade, representando diferentes tamanhos populacionais e semelhantes aos municípios brasileiros, com até 10 mil, 50 mil e mais de 100 mil mulheres, respectivamente.

Para assegurar a inexistência desses municípios, foi realizada uma consulta no sítio eletrônico do Instituto Brasileiro de Geografia e Estatística (IBGE), que lista todas as cidades brasileiras^(27)^. O planejamento e a execução do produto final ocorreram ao longo de aproximadamente quatro meses, de maio a agosto de 2022.

### Participantes

O estudo engloba um total de 160 mil participantes fictícias do sexo feminino, todas com vida sexual ativa e idades entre 25 e 64 anos. A personalização das participantes foi realizada utilizando *Fake Name Generator*™, uma ferramenta avançada para criar nomes e dados fictícios em 37 idiomas e para 31 países, ideal para gerar grandes volumes de dados fictícios para testes de desenvolvedores^(28)^.

No processo, foram selecionados nomes e sobrenomes de origens diversas, incluindo norte-americana, britânica, alemã, japonesa e espanhola, organizados em cinco planilhas individuais geradas pelo *Fake Name Generator*™. A compilação dessas planilhas produziu um banco de dados único, contendo 7.781 nomes, 16.860 sobrenomes, iniciais do segundo nome de A-Z e intervalo de datas de nascimento entre 27/07/1957 e 26/07/1997, abrangendo idades de 25 a 64 anos.

### Procedimentos e variáveis do estudo

As fontes de dados utilizadas para compor o modelo de painel digital de saúde foram baseadas em três relatórios quadrimestrais fictícios do SISAB, relativos ao primeiro quadrimestre de 2022, que contêm dados de mulheres entre 25 e 64 anos, de diferentes tamanhos populacionais. A estrutura dos bancos de dados fictícios foi alinhada com o formato do relatório do SISAB, incluindo seis colunas: “Nome”, Cadastro de Pessoa Física (CPF) “CPF”, Cartão Nacional do SUS (CNS) “CNS”, “Data de Nascimento”, “Última Coleta” e “Presente no Numerador”. Esta última coluna indica se a mulher realizou a coleta do exame citopatológico no período quadrimestral do relatório, com resposta “sim” ou “não”. Adicionalmente, foram inseridas duas novas colunas para registrar “Agendamento” (sim) e “Aprazamento” (dd/mm/aaaa), totalizando oito dimensões (variáveis).

A plataforma *Looker Studio* ^®^ (https://lookerstudio.google.com/) foi utilizada para a criação do modelo de painel digital de saúde^(26)^. Entre as várias opções de *softwares* disponíveis para criação de painéis, o *Looker Studio* ^®^ se destaca por ser uma ferramenta gratuita que transforma dados de relatórios em painéis interativos e de fácil compartilhamento, adaptáveis às necessidades específicas dos serviços de saúde^(22-23,26)^. Além disso, a solução digital facilita o armazenamento em nuvem, permitindo o acesso em tempo real desde que haja conexão à Internet.

Essa plataforma possibilitou a associação e dissociação de dados, bem como a criação de novas dimensões e recursos de filtragem para análise e gestão de informações. O *design* da interface foi fundamentado na Teoria das Cores^(29)^ e incluiu elementos de tipografia, logografia e gráficos de pizza, barras e linhas, para a comparação de dados e o uso de filtros. Para o período analisado, o modelo de painel digital utilizou dados simulados do primeiro quadrimestre de 2022, incluindo informações sobre novas coletas de exames citopatológicos realizadas, agendadas ou aprazadas, conforme o banco de dados fictício.

O cálculo do indicador “proporção de mulheres com coleta de citopatológico na APS”^(11)^ considera os seguintes elementos: “Numerador” – isto é, o número de mulheres com idade de 25 a 64 anos que realizaram o exame citopatológico na APS nos últimos três anos; “Denominador” – número de mulheres na mesma faixa etária cadastradas e vinculadas no mesmo território no período analisado ou potencial de cadastro municipal multiplicado pelo percentual de mulheres entre 25 e 64 anos, conforme estimativas populacionais de 2020 do IBGE; e o “Fator de multiplicação” – fixado em 100.

### Análise das simulações dos bancos de dados no modelo de painel de saúde

As simulações foram realizadas em três bancos de dados fictícios, formatados no *Google Sheets* ^®^ para representar diferentes tamanhos populacionais de mulheres entre 25 e 64 anos. Cada planilha incluía todas as dimensões (variáveis) mencionadas anteriormente, contendo dados sociodemográficos fictícios e aleatórios.

O modelo de painel de saúde foi submetido a testes de usabilidade, que envolveram a inserção dos diferentes bancos de dados fictícios durante reuniões remotas e sessões individuais. Esses testes avaliaram diversos aspectos, incluindo a facilidade de uso, a aparência visual, a interface interativa e a relevância do modelo de painel digital de saúde. Durante o desenvolvimento, buscou-se garantir que o painel digital fosse replicável e reutilizável por profissionais de saúde, especialmente enfermeiros, ao receberem os relatórios quadrimestrais do SISAB.

### Aspectos éticos

Este estudo dispensou a análise do Comitê de Ética em Pesquisa por se tratar do desenvolvimento de um produto tecnológico. Foram adotadas medidas para assegurar a conformidade com a Lei Geral de Proteção de Dados Pessoais (LGPD) nº 13.709/2018^(30)^ e 13.853/2019^(31)^. Para isso, relatórios quadrimestrais foram estruturados utilizando dados sociodemográficos fictícios de mulheres entre 25 e 64 anos, em municípios fictícios, no âmbito deste estudo de desenvolvimento tecnológico de um produto na área da saúde digital.

## Resultados

O presente estudo abordou a criação do modelo de painel digital de saúde destinado a enfermeiros, profissionais e gestores na APS, com o propósito de aprimorar a gestão de informações relacionadas ao indicador da proporção de mulheres que realizaram o exame citopatológico nos últimos três anos. O modelo foi desenvolvido para ser utilizado na unidade de saúde e reutilizado com dados reais dos relatórios quadrimestrais do SISAB. Os conjuntos de dados fictícios que serviram como fonte de dados podem ser acessados no endereço eletrônico https://bit.ly/BDcpPrevine, permitindo sua utilização em simulações.

### Organização do relatório quadrimestral em banco de dados (fonte de dados)

O relatório quadrimestral do SISAB, em formato de planilha, é uma fonte de informações detalhadas para a análise do indicador abordado neste estudo. Ele engloba variáveis relacionadas ao tempo, local e pessoa. Para transformar este relatório em um banco de dados utilizável (fonte de dados) é necessário padronizar os dados. Durante o tratamento, devem ser mantidas apenas as colunas com as informações “Nome” (coluna A), “CPF” (coluna B), “CNS” (coluna C), “Data de Nascimento” (coluna D), “Última Coleta” (coluna E) e “Presente no Numerador” (coluna F), assim como devem ser adicionadas mais duas colunas: “Agendamento” (coluna G) e “Aprazamento” (coluna H).

Os nomes das dimensões (variáveis) das colunas permaneceram inalterados, exceto pelas colunas adicionadas de “Agendamento” e “Aprazamento”, utilizadas para monitorar dados referentes à realização dos exames citopatológicos de forma oportuna ou organizada. Dessa forma, o modelo de painel digital pode reconhecer de maneira consistente o mesmo tipo de informação em qualquer banco de dados vinculado, considerando as diferenças de letras maiúsculas ou minúsculas, bem como a presença ou ausência de acentuação ortográfica. A Figura 1 oferece uma ilustração de um relatório quadrimestral fictício semelhante aos extraídos do SISAB.

**Figura 1:** - Tratamento do relatório quadrimestral fictício

É fundamental que o banco de dados seja constantemente atualizado pelo profissional de saúde, especialmente sob a liderança do enfermeiro, conforme as mulheres realizam o exame citopatológico durante o quadrimestre em andamento. Assim, o profissional de saúde responsável deve registrar a data da “Última Coleta” (coluna E) após a realização do exame citopatológico. No caso de agendamento confirmado (indicado como “sim”) com uma data de aprazamento (dd/mm/aaaa), a coluna “Última Coleta” deve ser atualizada após a realização do exame, e os dados das colunas “Agendamento” e “Aprazamento” devem ser removidos. Isso assegura que os resultados no painel de saúde se mantenham sempre atualizados, permitindo monitorar o progresso em direção à meta estabelecida pelo programa Previne Brasil.

### Vinculação do banco de dados ao *Looker Studio* ^®^

A Figura 2 detalha o processo de vinculação do banco de dados.

**Figura 2:** - Vinculação do banco de dados (fonte de dados) ao *Looker Studio* ^®^

Na etapa (1) “Fonte de dados”, é possível estabelecer conexões com diversos tipos de bancos de dados. Em seguida, na etapa (2), seleciona-se “Planilhas *Google*” e escolhe-se a opção (3) “Abrir a partir do *Google Drive*”. Na etapa subsequente (4), seleciona-se o banco de dados nomeado “BD_Alegria_do_Norte” e, por fim, realiza-se a conexão com o *Looker Studio* ^®^ na etapa (5). A Figura 3 explora as oito dimensões presentes na fonte de dados, permitindo a elaboração de novas fórmulas (fx).

**Figura 3:** - Dimensões (variáveis) do banco de dados (fonte de dados) ao *Looker Studio* ^®^

As fórmulas mencionadas anteriormente oferecem resultados simples, associações ou dissociações com o objetivo de consolidar informações para o modelo de painel digital de saúde. Antes da utilização do modelo, as nove fórmulas devem ser incorporadas à fonte de dados como novos “campos”, resultando em 17 dimensões (variáveis).

### Criação do modelo de painel de saúde

A criação do *design* de interface do modelo de painel digital foi uma abordagem inovadora para lidar com a complexidade da gestão da informação relacionada à proporção de mulheres que realizaram exame citopatológico. O *design* incorporou elementos originais da complexidade e heterogeneidade do público-alvo do indicador, destacando a interação com as mulheres como fundamental. Foram utilizados símbolos do Feminismo (roxo), conscientização do “Outubro Rosa” (rosa) e celebração do “Dia Internacional da Mulher” (lilás), com a aplicação da Teorias das Cores para o refinamento do *design* de interface final.

O modelo de painel digital recebeu o nome de Sistema de Informação em Saúde – Citopatológico (SIS-CP). Os resultados foram organizados em sete gráficos que combinam dados relevantes, permitindo uma análise detalhada da situação de saúde relacionada aos exames citopatológicos realizados em uma unidade de saúde e/ou município.

O painel digital pode ser utilizado por enfermeiros, profissionais e gestores de saúde para a gestão da informação. É importante destacar que tal painel não exibe dados sensíveis das mulheres, mas graficamente consolida dados em frequências absolutas, relativas e proporções de uma população fictícia das cidades simuladas neste estudo.

### Uso do modelo de painel de saúde

Para utilizar o painel SIS-CP, é necessário conectar o banco de dados ao modelo, disponível no endereço eletrônico: https://bit.ly/SISmeta4Previne. Após *download* do modelo, ele deve ser importado no *Looker Studio* ^®^ da unidade de saúde para visualizar os dados das cidades fictícias mencionadas anteriormente.

O SIS-CP foi composto por quatro telas. A primeira tela exibe a identificação da unidade de saúde e/ou município, seguida por três telas que apresentam os resultados. O tipo de gráfico foi selecionado de acordo com a variável em análise e sua configuração na fonte de dados, considerando período, dimensão, métrica e classificação no “*Setup*”. O painel é interativo, permitindo a exploração das informações com base nos filtros aplicados diretamente nos gráficos. A Figura 4 exemplifica o modelo de painel digital SIS-CP, utilizando dados da cidade fictícia de Alegria do Norte.

**Figura 4:** - Modelo de painel digital de saúde do Sistema de Informação em Saúde – Citopatológico (SIS-CP)

Na parte superior das três telas do painel SIS-CP, é exibido o nome do município fictício, que pode ser alterado manualmente. Também são apresentados três gráficos sob o título “Visão Geral” da aplicação *Looker Studio* ^®^. Estes gráficos representam: a frequência absoluta de mulheres entre 25 e 64 anos na população (“População”); a frequência absoluta de exames citopatológicos realizados (“Exames realizados”); e a frequência absoluta de exames citopatológicos agendados (“Exames agendados”).

Na segunda tela, o primeiro gráfico do painel SIS-CP (Figura 4) exibe um histograma com uma dispersão sobreposta para visualizar a distribuição de mulheres por faixa etária. O indicador “proporção de mulheres com coleta de citopatológico na APS” é representado no segundo gráfico (Figura 4) pela frequência relativa das mulheres que realizaram o exame citopatológico nos últimos três anos.

Na terceira tela, o terceiro gráfico (Figura 4) compara a frequência relativa das mulheres por faixa etária com a frequência relativa de exames citopatológicos coletados por faixa etária nos últimos três anos. O quarto gráfico (Figura 4) apresenta a frequência relativa de agendamentos de exames citopatológicos. A frequência relativa de 98,2% na figura indica dado “não registrado (NR)” na legenda, o que ocorre devido à ausência da descrição “sim” no banco de dados para as mulheres que realizaram a coleta do exame citopatológico e aquelas não agendadas pelo rastreamento oportunístico e/ou organizado.

Na última tela, o quinto gráfico (Figura 4) analisa a frequência de exames citopatológicos coletados, distribuídos por mês, buscando identificar a sazonalidade na realização dos exames para planejar estratégias de aumento da coleta de exames citopatológicos. O sexto gráfico (Figura 4) mostra a frequência relativa da realização do exame citopatológico nos três últimos anos, baseada na data da última coleta registrada. A frequência relativa de 75,9% no gráfico refere-se às mulheres sem agendamento e sem aprazamento para o exame citopatológico. A ausência da data de coleta efetiva do exame na coluna “Última Coleta” da fonte de dados indica um exame não realizado no período observado (NR). O último gráfico (Figura 4) projeta a quantidade de exames citopatológicos a serem realizados pelas mulheres a cada três anos, considerando a data da última coleta.

Para reutilizar o modelo, é essencial substituir o banco de dados na “Fonte de dados” do *Looker Studio* ^®^. O modelo de painel digital SIS-CP está disponível no endereço eletrônico https://bit.ly/BDcpPrevine, permitindo a simulação com bancos de dados fictícios deste estudo ou com dados reais. No caso de utilização do banco de dados proveniente do relatório quadrimestral do SISAB, ele deve ser organizado de forma que contenha as oito dimensões básicas mencionadas anteriormente. Em seguida, deve-se criar um relatório para o banco de dados recém-adicionado. Esse processo deve ser repetido a cada quadrimestre, quando o relatório é atualizado no SISAB. Essa etapa é fundamental para que o *Looker Studio* ^®^ reconheça o banco de dados como uma “Fonte de dados” e integre os dados ao painel digital SIS-CP.

A Figura 5 detalha o processo de substituição de bancos de dados e os ajustes mínimos necessários para (re)utilizar o modelo de painel digital. Esse procedimento pode ser conduzido sob a liderança do enfermeiro na APS para assegurar o correto funcionamento do painel digital de saúde, mantendo a proteção de dados sensíveis.

**Figura 5:** - Substituição de banco de dados no modelo de painel digital de saúde

Ao substituir a “Fonte de dados” no painel SIS-CP, a plataforma imediatamente sinaliza que (1) a configuração do gráfico está incompleta. Isso ocorre devido ao (2) filtro estabelecido para dados nulos (*null*) no banco de dados original, como exemplificado pelo “BD_Alegria_do_Norte”. Toda vez que um novo banco de dados é incorporado, a exemplo do “BD_Gentil_Flores”, o *software* identifica essa discrepância entre os bancos. Para corrigir a expressão dos dados, é necessário: (3) acrescentar um novo filtro no campo correspondente, destinado ao novo banco de dados, mantendo (4) o mesmo nome do filtro utilizado anteriormente. Após esse procedimento, (5) o filtro será reconhecido e o gráfico planejado no modelo será exibido com os dados corretos. Esse processo assegura que o painel digital SIS-CP funcione adequadamente com a nova “Fonte de dados”, garantindo a precisão e a integridade das informações apresentadas.

Adicionalmente, na Figura 5, é pertinente ressaltar que, durante a simulação com o banco de dados “BD_Nova_Felicidade”, observou-se a (6) expressão de uma pequena barra anterior às demais barras de faixas etárias estabelecidas nos gráficos das telas 2 e 3 do painel SIS-CP. Essas barras emergem devido à atualização contínua dos dados em tempo real. Como evidenciado (7), tal ocorrência deve-se ao fato de que 17 mulheres não mais se enquadram na faixa etária de 60 a 64 anos, tendo completado 65 anos durante o período do estudo. Esta observação assume importância ao tratar da avaliação de estratégias e da definição de prioridades específicas para a última faixa etária das mulheres.

O modelo de painel foi idealizado para ser reutilizado por profissionais de saúde, sobretudo enfermeiros em suas atividades na APS, bem como pela gestão municipal, proporcionando um acompanhamento em tempo quase real do indicador de proporção de mulheres com coleta de citopatológico. Importante ressaltar que o uso deste painel digital SIS-CP permite a atualização de dados de exames citopatológicos realizados como “Última Coleta” (dd/mm/aaaa), ou o preenchimento de “Agendamento” (sim) e “Aprazamento” (dd/mm/aaaa).

## Discussão

O desenvolvimento do modelo de painel digital SIS-CP representa uma inovação tecnológica promissora para a gestão da informação, proporcionando uma interface gráfica e interativa adaptada ao contexto atual das unidades da APS. Estudos anteriores na área da saúde infantil e neonatal^(22-23)^ destacaram funcionalidades e requisitos de interface que fomentam novas propostas para melhorar a gestão de informações e a tomada de decisões clínicas e administrativas.

De forma semelhante, este estudo discute a criação e reutilização do modelo de painel digital no contexto da APS, utilizando cenários de municípios fictícios para simulações alinhadas com a UX e aprendizado. Este modelo pode ser aplicado a bancos de dados reais provenientes dos relatórios do SISAB, transformando dados em informações de fácil acesso e interpretação.

O processamento e a análise de dados nas planilhas demandaram ferramentas robustas capazes de agregar informações em uma interface gráfica que auxilie profissionais de saúde na tomada de decisões. Durante a pandemia de COVID-19, isso se tornou ainda mais relevante para disponibilizar informações tanto para profissionais quanto para a população, além de promover uma participação mais ativa nos cuidados da saúde, utilizando abordagens do telemonitoramento, teleconsulta e telessaúde^(16,19-20)^.

Na área da saúde, a implementação de um sistema que emprega um painel digital de saúde pode ser altamente benéfica para auxiliar decisões gerenciais e assistenciais^(15,18,32-33)^. Isso possibilita uma análise detalhada da situação ao estabelecer conexões entre informações, identificar relações entre problemas de saúde e seus determinantes, avaliar riscos relacionados a doenças e elaborar estratégias futuras.

Essa abordagem alinha-se com a ideia de que iniciativas para melhorar o rastreamento do câncer de colo de útero devem ser compartilhadas^(34)^. Tanto enfermeiros quanto outros profissionais e gestores de saúde podem utilizar esse painel para a gestão da informação; entretanto, destacam-se a liderança e o protagonismo do enfermeiro^(35-36)^ na agilidade do tempo de resposta da equipe para definir e incentivar melhorias na qualidade dos serviços, alinhando-se com as políticas públicas de saúde da mulher e na gestão de recursos financeiros na APS.

Na Austrália, um estudo utilizou dados dos serviços de saúde mental para realizar ajustes pontuais durante a pandemia de COVID-19, abrangendo várias esferas político-administrativas, implementando ações mais eficazes e acordos cooperativos para compartilhamento de dados^(19)^. Esse exemplo reforça a importância dos painéis digitais de saúde, que oferecem análise situacional em tempo quase real^(21)^, permitindo a proposição de estratégias concretas baseadas em dados epidemiológicos.

O sucesso da tomada de decisão baseia-se na qualidade das informações acessadas por profissionais e gestores de saúde^(37-38)^. A análise visual facilita o raciocínio analítico de dados abstratos por meio de uma boa interface gráfica^(32,39)^. A adoção de painéis digitais de saúde valoriza a produção de cuidado^(40)^, oferecendo uma visão abrangente de padrões, tendências e correlações de resultados^(38)^ em tempo real^(8,41)^.

Em outro estudo, dados extraídos dos prontuários eletrônicos de usuários hipertensos na APS resultaram na construção de um painel que demonstrou sua utilidade no fluxo de trabalho dos profissionais de saúde. Alinhando as informações com as diretrizes clínicas, esse painel otimizou o tempo e qualificou as consultas^(39)^. Um estudo adicional observou que o painel criado pelo *Medicare* ^®^ para promover equidade na saúde revelou um desempenho limitado em relação ao acesso aos serviços de saúde, permitindo a supervisão e a priorização de esforços para melhorar a acessibilidade^(15)^.

Por outro lado, um estudo que mapeou as principais fontes de dados para um painel de monitoramento e avaliação da gestão do SUS concluiu que ainda há um desafio significativo devido à fragmentação dos sistemas de informação no Brasil. No entanto, os pesquisadores relativizaram essa questão, afirmando que encontrar instrumentos e fontes de informação de acesso rápido e fácil pode viabilizar a avaliação e monitoramento de saúde em situações específicas^(40)^.

Assim, os painéis digitais de saúde demonstram a capacidade de analisar e comunicar uma variedade de informações relevantes^(14,33)^ para enfermeiros e profissionais de saúde que atuam na APS, por meio do aproveitamento dos relatórios do SISAB. Essa abordagem possibilita um monitoramento dinâmico e atualizado do indicador, contribuindo para a gestão de informações na APS.

O modelo de painel digital pode servir como base para orientar o processo de planejamento, monitoramento e avaliação de intervenções, contribuindo para a melhoria da acessibilidade, do desempenho e da qualidade dos serviços de saúde oferecidos às mulheres. Um estudo avaliando ações de controle do câncer de colo de útero, com dados registrados de 2013 a 2020, exemplifica essa aplicação^(42)^. Outro estudo sobre o impacto da pandemia de COVID-19 na realização do exame preventivo do câncer de colo de útero reforçou a importância de ações e estratégias eficazes para aumentar a adesão e incentivar as mulheres^(41)^ a assumir o protagonismo no cuidado da saúde.

Este estudo adotou uma abordagem social e ética, com o propósito de contribuir para os setores da saúde, educação e sociedade. O modelo de painel digital e bancos de dados fictícios estão disponíveis gratuitamente, permitindo simulações com os relatórios quadrimestrais reais do SISAB de qualquer unidade de saúde e/ou município. Nessa perspectiva, uma pesquisa sobre conhecimentos, atitudes e práticas de 170 profissionais de saúde pertencentes a 94 equipes de saúde de um município de Minas Gerais enfatizou a necessidade de ações de educação permanente para enfermeiros e médicos na APS em relação ao câncer de colo de útero^(43)^.

Sobre as limitações do uso do painel digital de saúde SIS-CP, observa-se uma restrição relacionada ao período específico (quadrimestre), requerendo que o banco de dados seja atualizado e reorganizado a cada quatro meses. Outra limitação é a exclusão de dados de mulheres com menos de 25 anos ou mais de 65 anos, conforme estipulado pelo indicador para o rastreamento de câncer de colo de útero. Além disso, há uma escassez de registros sobre a adoção e implementação de novas tecnologias para a gestão da informação por enfermeiros, profissionais de saúde e gestores no âmbito do SUS.

Este estudo tem a capacidade de instigar futuras investigações e o desenvolvimento de mecanismos destinados ao planejamento, ao monitoramento e à avaliação na área da saúde. Também oferece oportunidades de aprimorar o painel digital SIS-CP por meio da UX, visando tornar as informações mais compreensíveis e acessíveis para diferentes níveis de letramento e necessidades de saúde. O modelo de painel digital de saúde pode ser aplicado em simulações durante a formação acadêmica de enfermeiros e profissionais da área da saúde, ampliando a oportunidade de aprimorar as habilidades no uso de tecnologias, como o *Looker Studio* ^®^, para criar e aperfeiçoar novos painéis digitais de saúde.

## Conclusão

O estudo destaca a relevância e a inovação proporcionada pela criação de um modelo de painel digital de saúde SIS-CP, uma solução digital avançada para a gestão de informações na APS. Este modelo sobressai-se pela interface gráfica interativa e pela capacidade de fornecer aos enfermeiros, profissionais e gestores de saúde uma ferramenta eficaz para planejar a assistência à saúde das mulheres entre 25 e 64 anos, monitorar a realização do exame citopatológico, rastrear o câncer de colo de útero e avaliar acessibilidade e qualidade assistencial por meio do indicador para atingimento da meta no programa Previne Brasil.

A adoção da tecnologia nas práticas diárias permite aos profissionais um planejamento personalizado que atende às necessidades específicas da população feminina de uma região delimitada. Assim, a ferramenta não apenas apoia a tomada de decisão, gestão da informação e financiamento do SUS, mas contribui para ampliar o número de mulheres que realizam a coleta de citopatológico. Com essa ação preventiva, é possível tratar lesões precocemente, aumentando a sobrevida e reduzindo mortes prematuras causadas pelo câncer de colo de útero. Portanto, o painel digital SIS-CP destaca-se como um importante aliado no rastreamento do câncer de útero e na promoção da saúde das mulheres.
